# Changes in striatal dopamine release, sleep, and behavior during spontaneous Δ-9-tetrahydrocannabinol abstinence in male and female mice

**DOI:** 10.1038/s41386-022-01326-0

**Published:** 2022-04-27

**Authors:** Andrew J. Kesner, Yolanda Mateo, Karina P. Abrahao, Stephanie Ramos-Maciel, Matthew J. Pava, Alexa L. Gracias, Riley T. Paulsen, Hartley B. Carlson, David M. Lovinger

**Affiliations:** 1 National Institute on Alcohol Abuse and Alcoholism, Intramural Research Program, NIH, Bethesda, MD USA; 2 Center on Compulsive Behaviors, Intramural Research Program, NIH, Bethesda, MD USA; 3 Departamento de Psicobiologia, Universidade Federal de São Paulo, Campus São Paulo, São Paulo, SP Brazil; 4 Arlington, VA USA

**Keywords:** Reward, Sleep, Motivation

## Abstract

Withdrawal symptoms are observed upon cessation of cannabis use in humans. Although animal studies have examined withdrawal symptoms following exposure to delta-9-tetrahydrocannabinol (THC), difficulties in obtaining objective measures of spontaneous withdrawal using paradigms that mimic cessation of use in humans have slowed research. The neuromodulator dopamine (DA) is affected by chronic THC treatment and plays a role in many behaviors related to human THC withdrawal symptoms. These symptoms include sleep disturbances that often drive relapse, and emotional behaviors like irritability and anhedonia. We examined THC withdrawal-induced changes in striatal DA release and the extent to which sleep disruption and behavioral maladaptation manifest during abstinence in a mouse model of chronic THC exposure. Using a THC treatment regimen known to produce tolerance, we measured electrically elicited DA release in acute brain slices from different striatal subregions during early and late THC abstinence. Long-term polysomnographic recordings from mice were used to assess vigilance state and sleep architecture before, during, and after THC treatment. We additionally assessed how behaviors that model human withdrawal symptoms are altered by chronic THC treatment in early and late abstinence. We detected altered striatal DA release, sleep disturbances that mimic clinical observations, and behavioral maladaptation in mice following tolerance to THC. Altered striatal DA release, sleep, and affect-related behaviors associated with spontaneous THC abstinence were more consistently observed in male mice. These findings provide a foundation for preclinical study of directly translatable non-precipitated THC withdrawal symptoms and the neural mechanisms that affect them.

## Introduction

### Cannabis use and abuse

Cannabis derivatives are currently the most widely used illicit drugs in the world [1], with reported use increasing, at least in part, due to efforts towards its legalization [2]. While adverse effects of cannabis use are often mild, a subpopulation of chronic cannabis users experience hallmarks of a substance use disorder (SUD), including use despite adverse consequences, craving, tolerance to the drug’s effects, and withdrawal symptoms [3–5]. Experiencing these symptoms can lead to diagnosis of cannabis use disorder (CUD) as defined by the DSM-5 [6].

The main psychoactive component of cannabis is Δ-9-tetrahydrocannabinol (THC). This compound is primarily responsible for the drug-induced “high” via actions at the cannabinoid-type 1 receptor (CB1) [7, 8]. Indeed, behavioral effects of chronic THC treatment are absent in mice genetically engineered to lack the CB1 receptor [9, 10], and CB1 antagonists prevent self-administration of synthetic cannabinoids [10, 11].

### Cannabis withdrawal symptoms

Cessation of chronic cannabis or THC use causes withdrawal symptoms in a significant population of users [12–25]. In humans, cannabis withdrawal symptoms (CWS) may include: irritability/aggression, nervousness/anxiety, disrupted sleep, hypophagia and weight loss, restlessness, depressed mood, and uncomfortable somatic symptoms e.g., abdominal pain, shakes, sweating, fever/chills, or headache [6]. One of the most prominent CWS is disrupted sleep, and poor sleep quality is a major risk factor towards cannabis relapse [26–28]. Human studies show that acute THC produces sleep facilitation including shorter sleep latency, less difficulty falling asleep and more time spent asleep [29–32]. Our group has shown that endocannabinoid (eCB) activity contributes to non-rapid eye movement, (NREM) stability [33]. The sleep-inducing effects of acute THC may dissipate during chronic exposure in humans [29], and abstaining from chronic THC or cannabis use in humans is associated with decreased sleep time and efficiency [34–36].

At the preclinical level, while some studies have modeled CWS using spontaneous withdrawal (abrupt cessation of drug treatment), the far more prevalent model is withdrawal precipitated by CB1 antagonist/inverse agonist administration, which immediately blocks the effects of the chronically administered cannabinoid drug [37]. An obvious caveat of precipitated withdrawal is that humans undergo spontaneous withdrawal, so the translational relevance of precipitated CWS is unclear. While spontaneous cannabis or THC withdrawal does reliably induce somatic withdrawal symptoms (e.g., wet dog and head shaking, front paw tremor, hunched posture, body tremor, etc.) [38–41], indicating its potential to drive CWS, previous studies have struggled to provide strong evidence for spontaneous THC withdrawal symptoms that more closely model human CWS.

### Dopamine, CUD and CWS

The neuromodulator dopamine (DA) has well established roles in SUDs [42, 43], behavioral alterations during drug withdrawal [44, 45], and sleep [46–48]. There is considerable evidence that exogenous cannabinoids indirectly modulate DA activity [49–51] with ramifications for development of CUD and CWS. The striatum, comprised of the nucleus accumbens (NAc), dorsal medial (DMS) and dorsal lateral striatal (DLS) subregions, is a major site of DA action in the brain and is involved in many behaviors associated with drug abuse and withdrawal symptoms [52], as well as sleep [53], making it an interesting nexus THC action and withdrawal phenomena.

### Aim of study

In the present study, we modeled chronic cannabis use using a well established procedure to induce THC tolerance in mice [54] to examine whether spontaneous THC withdrawal drives: (1) alterations in striatal DA release, (2) sleep disruption, and (3) translationally relevant behavioral maladaptation. To examine effects of acute and chronic THC treatment on behavior and sleep, we compared vehicle injections to the first and last injections of the chronic THC or vehicle control (VEH) treatment. To examine maladaptive behavior and sleep during withdrawal, we compared changes in baseline metrics (2 days pretreatment) to early (1–2 days) and late (5–6 days) abstinence in mice treated with THC or VEH (Supplementary Fig. 1). Both male and female mice were examined independently, as accumulating evidence suggests sex differences in CUD and severity of CWS [55].

## Methods and materials

### Subjects

All experiments were conducted in the same facility at the National Institute on Alcohol Abuse and Alcoholism using 8 to 10-week-old wildtype male and female mice (Mus musculus, C57BL/6 J strain) at the time of electroencephalogram/electromyogram (EEG/EMG) implantation or behavioral studies. For full description of subjects see supplementary information. All methods used in this work were approved by the Animal Care and Use Committee of the National Institute on Alcohol Abuse and Alcoholism (protocol #: LIN-DL-1) and were within the guidelines described in the NIH Guide to the Care and Use of Laboratory Animals. Note that all times of day are expressed in terms of hours from lights on, i.e., zeitgeber time (ZT). Separate cohorts of mice were used in all experiments.

### Drugs

THC stock solution (100 mg/mL in ethanol) was obtained from the U.S. National Institute on Drug Abuse. Ethanol was evaporated using nitrogen gas and the remaining THC was re-dissolved in dimethysulfoxide (DMSO) and aliquoted for storage at −20^C^. THC at the various doses used in these studies was made fresh before each administration in a vehicle consisting of 1:1:18 (DMSO : Cremaphor : 0.9% Saline) (Cat# D2650 and C5135 for DMSO and Cremaphor EL, respectively, Sigma Aldrich, St. Louis, MO).

### Sleep recordings

Sleep experiments were performed as previously described [33, 56]. See supplementary information for full description of surgical procedures, recording environment, polysomnographic acquisition, and vigilance state scoring (e.g., NREM vs REM sleep).

### THC treatment and behavioral paradigms

#### Sleep effects during and after THC treatment

Recordings and treatment began after the 7-day acclimatization period. First, we collected 2 separate days of baseline polysomnographic measurements (pretreatment). Following stoppage of recording on the 2nd day of baseline, all mice received injection of VEH. After stoppage of recording for this VEH treatment, mice were treated with either VEH or 10 mg/kg of THC in vehicle and EEG/EMG were recorded for another ~22 h. After stoppage of recording for the first injection, the animals received injections according to their assigned group twice daily for four more days – once before the dark phase, between ZT11:00 and ZT12:00, and the second injection between ZT1:00 and ZT2:00. Treatments were assigned randomly such that each recording chamber with five individually housed mice had roughly half the subjects receiving either THC or VEH. Injections for Vehicle-only session and first injection occurred within the last hour before the start of the dark phase, between ZT11:00 and ZT12:00. Finally, the mice received one last injection, again between ZT11:00 and ZT12:00, and EEG/EMG were recorded for ~22 h. EEG/EMG recording continued for the next 6 full days of abstinence (ABST 1–6). Each ABST recording began between ZT11:00 and ZT12:00, and lasted ~22 h. To measure effects from chronic low dose THC, the same procedure as above was used, but a 1 mg/kg dose of THC was given. To measure sleep effects during abstinence from an acute dose of THC, the same procedure as chronic 10 mg/kg THC experiments was used except a single THC treatment was immediately followed by ABST 1–6.

#### Behavioral effects of chronic 10 mg/kg dose of THC

For all awake behaviors, mice were treated with the chronic 10 mg/kg dose of THC using the treatment procedure described above in sleep experiments. All mice were initially group housed, four animals per cage. Before all experiments mice were transported to respective behavioral rooms and handled daily for 3 days.

See supplementary information for full description of sucrose preference test, operant conditioned sucrose seeking, intake and locomotor activity metrics, and bottle brush test procedures.

### Fast scan cyclic voltammetry

Following isoflurane anesthesia, brains were removed and 300 µm-thick coronal sections through the striatum were prepared (Leica VT1200S, Leica Biosystems, IL) in ice-cold carbogen-saturated (95% O_2_/5% CO_2_) cutting solution (in mM: Sucrose 194, NaCl 30, KCl 4.5, MgCl_2_ 1, NaHCO_3_ 26, NaH_2_PO_4_ 1.2, Glucose 10). Slices were then transferred to a chamber filled with oxygenated artificial cerebrospinal fluid (aCSF) (pH 7.4) containing (in mM): NaCl (126), KCl (2.5), NaHCO_3_ (25), NaH_2_PO_4_ (1.2), dextrose (10), HEPES (20), CaCl_2_ (2.4), MgCl_2_ (1.2), and L-ascorbic acid (0.4) kept at 32 °C and allowed to recover for 1 h until used for recordings. After the equilibration period, brain slices were transferred to the recording chamber and perfused at a rate of ~1.5 mL/min with aCSF. Once the brain slice was in place, a bipolar stainless-steel stimulating electrode (Plastics One, Roanoke, VA) was placed in the region of interest and a carbon fiber electrode was placed ~300 µm from the stimulating electrode. Cylindrical carbon fibers (T650 carbon fiber, 7 μm diameter, 100–150 μm exposed length; Goodfellow, PA) were inserted into a glass pipette [57]. The carbon fiber electrode was held at −0.4 V, and the potential was increased to 1.2 V and back at 400 V/s every 100 ms using a triangle waveform. DA release was evoked by rectangular, electrical pulse stimulation (250–800 µA; 2 ms, monophasic) applied every 5 min with a NL 800 A Current Stimulus Isolator (Digitimer, Hertfordshire, UK). Data collection and analysis were performed using the Demon Voltammetry and Analysis software suite [58]. The maximum amplitudes of extracellular DA transients were obtained from input/output (I/O) curves constructed by plotting stimulus current versus peak DA concentration calculated from the peak amplitude of the stimulus-induced transient over a range of stimulus intensities measured in three areas of the striatum: DLS, DMS, and NAc. Carbon fiber electrodes were calibrated using 1.0 uM DA after recordings. All tissue was harvested between ZT2:00 – ZT4:00 on the abstinence day of interest. When possible, all three regions of interest were measured within each slice across animals.

### Plasma corticosterone quantification

Blood was collected via lateral tail vein bleeding between ZT10-12. See supplementary information for full description of plasma corticosterone collection procedures. Plasma corticosterone concentration was determined using enzyme-linked immunosorbent assay as per manufacturer’s instructions for small sample volume (ELISA part # ADI-901-097, Enzo Life Sciences, Inc., NY). The assay was performed in duplicate and read using a SpectraMax190 Microplate Reader. Standard curves and corticosterone concentration extrapolation were performed using an open-access online analysis service (MyAssays Ltd., USA).

### Statistical analysis

Data were analyzed using GraphPad Prism (version 9; GraphPad Software, La Jolla, CA, USA) statistics software. All behavioral data and CORT measurements were transformed to change, “Δ”, from vehicle injection (when investigating effects of first and last treatments) or pretreatment (when investigating effects during early and late abstinence). For sleep, intake/locomotion, sucrose preference test, and operant tasks, the pretreatment, early, and late withdrawal metrics were the average of the  2 pretreatment sessions, first 2, and last 2 days of abstinence sessions, respectively. For all other experiments we only recorded metrics on one pretreatment day and the 2nd and 6th days of abstinence, so analysis reflects those single sessions. Sex differences are known to exist in most of the metrics we examine, so we decided, a priori, to analyze male and female mice independently. See supplementary information for full description of statistical analysis.

## Results

### Striatal dopamine release in early and late abstinence after acute and chronic THC treatment

#### Changes in DA release during abstinence following acute THC treatment

We first used fast scan cyclic voltammetry to measure electrically stimulated DA release in the NAc, DMS, and DLS during early and late abstinence following acute THC treatment. Male mice showed a significant increase in peak DA release in DMS following a single, i.e., acute, injection of THC (Supplementary Fig. 2A). This increase was observed during early (Supplementary Fig. 2B) and late abstinence (Supplementary Fig. 2C). However, there were no differences in peak DA release in the DLS or NAc after an acute THC injection. In contrast, female mice showed decreased peak DA levels in DMS and NAc following a single THC dose in early abstinence. However, this same THC administration protocol elicited an increase in peak DA levels in DLS (Supplementary Fig. 2D). In late abstinence, after acute THC treatment, no differences in peak DA release were observed (Supplementary Fig. 2E). THC treatments did not elicit changes in uptake rate (data not shown).

#### Changes in DA release during abstinence following chronic THC treatment

**Fig. 1:**
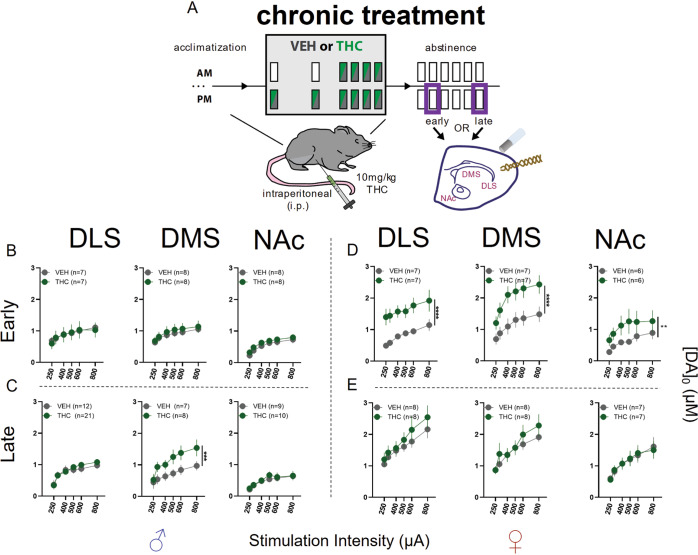
Effects of chronic THC treatment on striatal DA release in acute brain slices using FSCV. **A** Schematic illustrating chronic treatment and timepoints of FSCV recordings in striatal subregions where a carbon fiber recording electrode and stimulating electrode were placed to record electrical stimulation-elicited DA release. Purple rectangles indicate abstinence timepoints used in recordings. **B** DA release in males (♂) during early abstinence following chronic treatment. No differences detected in DLS (F_treatment_(1,72) = 0.02, *p* = 0.8712), DMS (F_treatment_(1,84) = 1.01, *p* = 0.3168), or NAc (F_treatment_(1,84) = 3.07, *p* = 0.0834). **C** DA release in males during late abstinence following chronic treatment. No differences detected in DLS (F_treatment_(1,186) = 0.45, *p* = 0.5019) or NAc (F_treatment_(1,87) = 0.64, *p* = 0.4270). In DMS THC treated mice had elevated DA release (F_treatment_ (1,71) = 13.56; *p* = 0.0004). **D** DA release in females (♀) during early abstinence following chronic treatment. DA release was elevated in DLS (F_treatment_(1,72) = 51.61, *p* = <0.0001), DMS (F_treatment_(1,72) = 33.82, *p* = <0.0001) and NAc (F_treatment_(1,60) = 11.69, *p* = 0.0011). **E** DA release in females during late abstinence following acute treatment. No differences detected in DLS (F_treatment_(1,84) = 2.54, *p* = 0.1151), DMS (F_treatment_(1,84) = 1.68, *p* = 0.1981), or NAc (F_treatment_(1,72) = <0.01, *p* = 0.9976).

In male mice exposed to chronic THC treatment (Fig. 1A), a significant increase in DMS DA release was present during late abstinence but not early abstinence (Fig. 1B, C). In females, during early abstinence we found a robust increase in DA levels in all three striatal regions (Fig. 1D), but no such increase during late abstinence (Fig. 1E).

### Sleep and vigilance state architecture during acute and chronic THC treatment and abstinence

We next examined if THC and VEH treatments changed percent time spent in NREM and REM sleep and sleep architecture (i.e., bout number and duration) referenced to baseline metrics (i.e., vehicle treatment or pretreatment epochs). Note that the sleep and behavioral data that follow are reported as a change-score (i.e., delta “∆”) from either vehicle treatment or pretreatment epochs. Additionally, we refer to light-photoperiod as lights on (LON) and dark-photoperiod as lights off (LOFF) throughout the remainder of this report.

#### Acute and chronic THC administration alters sleep in both sexes

We first examined how VEH or THC treatments altered sleep after the first and last injections of the chronic treatment regimen compared to the vehicle treatment session (Supplementary Fig. 3A, B). In male mice, the first THC injection increased percent time spent in NREM compared to VEH during LOFF. After the last injection, percent time in NREM was lower in THC treated compared to VEH treated male mice during both LOFF and LON. During LOFF, THC treated male mice showed a lesser change in percent time in NREM as compared to the first injection (Supplementary Fig. 3C). No significant effects were detected for NREM bout duration (Supplementary Fig. 3D). However, an increased number of NREM bouts was observed during LOFF in THC treated male mice compared to VEH treated mice. After the last injection, the number of NREM bouts during both LOFF and LON was decreased regardless of treatment when compared to the first injection (Supplementary Fig. 3E).

Small changes in percent time in REM sleep were observed following the first THC injection, but post-hoc testing found no significant differences between treatments or within treatment groups across epochs (Supplementary Fig. 3F). In contrast, after the last injection, THC treated male mice showed larger increases in REM bout duration compared to VEH treated mice during LOFF. THC treated male mice also had longer REM bout durations after the last injection compared to first injection during both photoperiods (Supplementary Fig. 3G). THC treated male mice also exhibited increased number of REM bouts during LOFF after the first injection compared to VEH treated mice, while the number of REM bouts in THC treated mice decreased after the last injection when compared to the first injection for both LOFF and LON (Supplementary Fig. 3H).

Female mice also exhibited increased percent NREM sleep after acute THC (Supplementary Fig. 3I), that was abolished by the end of chronic treatment. In contrast to male mice, NREM sleep time did not differ between THC and VEH treated females at the end of chronic treatment. Neither acute nor chronic THC administration altered NREM bout duration in female mice (Supplementary Fig. 3J). While there was an overall increase in the number of NREM bouts, there were no pair-wise differences between groups (Supplementary Fig. 3K). Also, in contrast to male mice, we observed no effect of THC injections on REM sleep time or architecture (Supplementary Fig. 3L-N).

#### Chronic THC transiently disrupts sleep in male mice at early abstinence timepoints

**Fig. 2:**
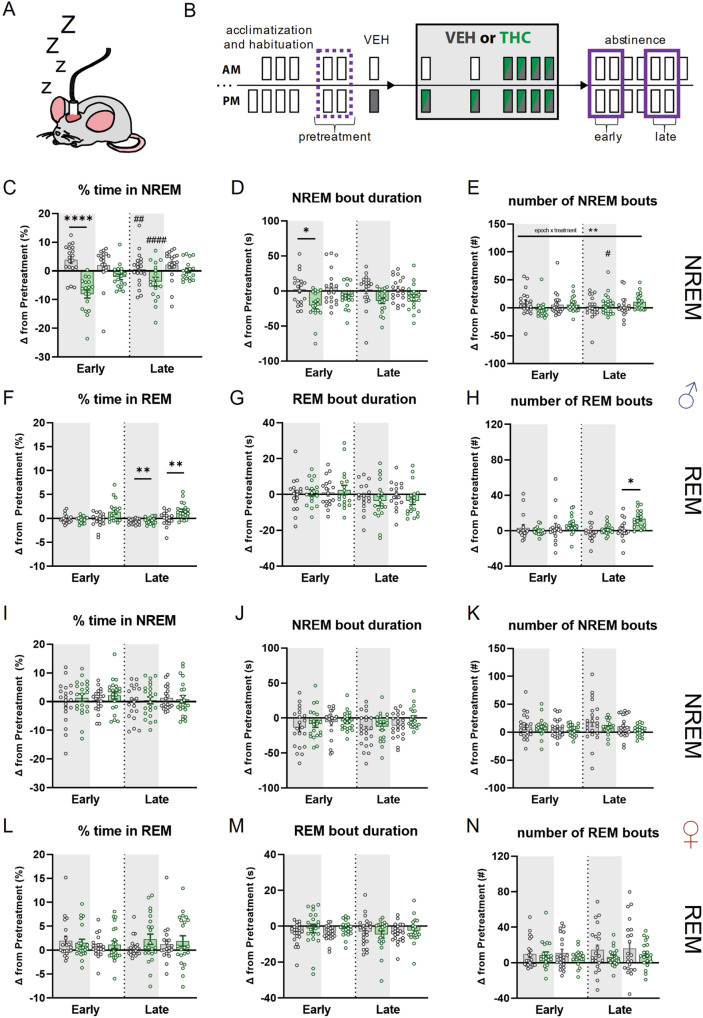
Effects of chronic THC administration on sleep in male and female mice during early and late abstinence. **A** Cartoon illustrating chronically tethered mouse during epochs where no injection is given, i.e., pretreatment and abstinence. **B** Timeline illustrating treatment regimen and epochs contributing to analysis. Dotted purple line indicates average of two pretreatment epochs that average of days 1 and 2 of early and days 5 and 6 of late abstinence are compared. **C** Comparison between effects of THC or VEH on changes from pretreatment in percent time spent in NREM sleep in male (♂) mice. There is a significant treatment effect (F_treatment_(1,35) = 20.64; *p* = <0.0001), with post-hoc tests finding differences between-groups during Early-LOFF (p**** = <0.0001). Both groups had significant within group difference between Early and Late abstinence LOFF (THC *p*^####^ = <0.0001; VEH *p*^##^ = 0.0030). **D** Comparison between effects of THC or VEH on changes from pretreatment in NREM bout duration in male mice. There is a significant treatment effect (F_treatment_(1,35) = 11.14; *p* = 0.0020), with post-hoc tests finding differences between-groups during Early-LOFF (p* = 0.0113). **E** Comparison between effects of THC or VEH on changes from pretreatment in number of NREM bouts in male mice. There is a significant interaction (F_epoch×treatment_(1,37) = 12.64; *p* = 0.0011). **F** Comparison between effects of THC or VEH on changes from pretreatment in percent time spent in REM sleep in male mice. There is a significant treatment effect (F_treatment_(1,33) = 8.048; *p* = 0.0077), with post-hoc tests finding differences between-groups during Late-LOFF (p** = 0.0017) and Late-LON (p** = 0.0057). **G** Comparison between effects of THC or VEH on changes from vehicle injection in REM bout duration in male mice. No significant effects were detected (F_epoch×treatment_(1,33) = 3.447; *p* = 0.0732). **H** Comparison between effects of THC or VEH on changes from pretreatment in number of NREM bouts in male mice. There is a significant interaction (F_epoch×treatment_(1,33) = 5.744; *p* = 0.0224), with post-hoc tests finding differences between-groups during Late-LOFF (p* = 0.0228). (**I**–**N**) Same metrics as (**C–H**) but in female (♀) mice. No significant effects were detected. **I** F_epoch×treatment_(1,42) = 1.191; p = 0.2814. **J** F_lights×epoch×treatment_(1,33) = 3.881; *p* = 0.0581. **K** F_epoch×treatment_(1,42) = 6.279; *p* = 0.4326. **L** F_lights×epoch×treatment_ (1,30) = 3.260; *p* = 0.0810. **M** F_epoch×treatment_(1,42) = 3.649; *p* = 0.0630. **N** F_lights×epoch×treatment_(1,42) = 1.816; *p* = 0.1879.

Because sleep disturbances are commonly reported by individuals with CUD during abstinence [35, 59, 60], we obtained electrographic measures of sleep after the 6-day chronic treatment regimen and normalized these to the pretreatment baseline for each subject (Fig. 2A, B). In male mice, we observed reduced NREM sleep following chronic THC treatment, evidence of acute, spontaneous withdrawal symptoms in early abstinence (Fig. 2C). These effects were specific to LOFF in early abstinence, and they recovered by late abstinence, when THC treated males slept no differently than the VEH group. Reduced sleep during early abstinence was largely due to a reduction in the duration of NREM bouts with THC treated subjects exhibiting decreased bout duration during LOFF in early but not late abstinence (Fig. 2D). While interaction effects for NREM bout number were observed in late-LOFF compared to early-LOFF in male THC treated mice, post-hoc tests found no significant differences related to treatment (Fig. 2E).

Chronic THC treatment in male mice led to increased time spent in REM sleep compared to VEH treatment in LOFF and LON periods late in abstinence (Fig. 2F). We did not detect any treatment related effects on REM bout duration (Fig. 2G). THC treated male mice had greater increases in REM bout number compared to VEH treated mice during LOFF in late abstinence (Fig. 2H).

In contrast to male mice, no significant treatment related main or interaction effects were detected for NREM or REM metrics during abstinence in females (Fig. 2I–N).

#### Low dose chronic treatment does not strongly alter sleep during administration or abstinence

We next performed separate experiments where mice were chronically treated with a lower dose (1.0 mg/kg) of THC. (Supplementary Fig. 4A, B). During the treatment period we only detected a decrease in NREM bout duration and number in THC treated relative to VEH treated mice during LON after the last injection (Supplementary Fig. 4C–E). A significant interaction in number of REM bouts was detected, but no significant differences between treatments or across injection epochs was found in post-hoc tests (Supplementary Fig. 4F–H). We found no significant main or interaction effects of low dose chronic THC treatment in female mice for NREM sleep metrics, nor percent time in REM or number of REM bouts. We did detect an interaction effect on REM bout duration in these groups, but post-hoc tests found no significant treatment related effects (Supplementary Fig. 4I–K). Low dose chronic THC treatment did not alter any sleep metrics during early or late abstinence when compared to the pretreatment epoch in either sex (Supplementary Fig. 5A–N).

#### Acute THC treatment does not alter sleep during abstinence

Profound sleep disturbances were only observed in male mice after chronic 10 mg/kg THC treatment. These changes could be due to effects of the final THC treatment in the chronic regimen rather than changes accrued during chronic treatment. To examine this possibility, we gave male mice a single 10 mg/kg THC treatment and examined sleep in abstinence. A significant interaction was detected for changes in NREM and REM bout duration, but no significant differences between treatments or across abstinence epochs were observed (Supplementary Fig. 6A–H). Thus, the effects of chronic 10 mg/kg THC in male mice are likely due to accrued effects of chronic treatment.

### Sustenance intake and locomotor activity during chronic THC treatment and abstinence

**Fig. 3:**
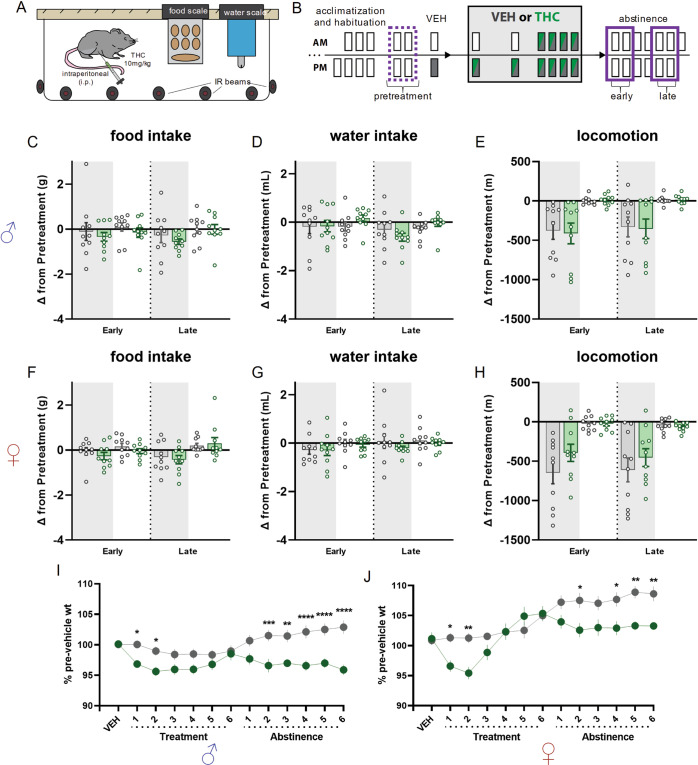
Measurements of changes in food and water intake, and locomotion, during early and late abstinence. **A** Cartoon illustrating home cage intake and locomotion monitoring platform. **B** Timeline illustrating treatment regimen and epochs contributing to analysis. Dotted purple line indicates average of two pretreatment epochs that average of days 1 and 2 of early and days 5 and 6 of late abstinence are compared. **C**–**E** Comparison between effects of THC or VEH on changes from pretreatment during early and late abstinence epochs in food and water intake, and locomotion in male (♂) mice. No significant effects were detected. **C** F_lights×epoch×treatment_ (1,18) = 0.7414; *p* = 0.4005. **D** F_lights×treatment_ (1,18) = 3.369; *p* = 0.0830. F_lights×epoch×treatment_ (1,18) = 1.514; *p* = 0.7018. **F**–**H** Same as (**C**–**E**) for female mice (♀). No significant effects were detected. **F** F_epoch×treatment_ (1,18) = 1.201; *p* = 0.2876. **G** F_lights×epoch×treatment_ (1,18) = 0.8053; *p* = 0.3814. **H** F_epoch×treatment_ (1,18) = 1.295; *p* = 0.1041. **I** Percent of Vehicle treatment weights during chronic THC treatment and abstinence in male mice. There is a significant treatment effect (F_treatment_(1,18) = 11.08; *p* = 0.0037), with post-hoc tests finding differences between-groups (p* = <0.05, p** = < 0.01, p*** = < 0.001, p**** = < 0.0001). **J** Percent of Vehicle treatment weights during chronic THC treatment and abstinence in female mice. There is a significant treatment effect (F_treatment_(1,18) = 5.577; *p* = 0.0297), with post-hoc tests finding differences between-groups (p* = < 0.05, p** = < 0.01).

Accumulating evidence indicates humans experience altered hunger and thirst during cannabis withdrawal. These symptoms are often accompanied by restlessness which might manifest as increased locomotor behavior in general contexts. Thus, we examined how food and water intake and locomotor behavior are altered during early and late abstinence following chronic THC treatment in mice (Supplementary Fig. 7A, B). For both males and females, significant treatment related effects were detected for food and water intake, and locomotion (Supplementary Fig. 7C–H). Additionally, THC treated animals showed decreased food and water intake during LOFF after the first injection of the chronic treatment regimen, which differed from VEH treated mice. Changes from vehicle treatment did not differ between groups after the last chronic treatment, but after the last injection both sexes showed normalization of intake back towards vehicle treatment levels during LOFF. Similarly, THC treated mice of both sexes had reduced changes in locomotion compared to VEH treated mice during LOFF after the first treatment. While VEH treated mice showed reduced locomotion after the last treatment compared to first treatment, an increase was found in THC treated mice. THC treatment did not alter food or water intake and locomotion during early or late abstinence when compared to the pretreatment epoch in either sex (Fig. 3A–H).

THC treated mice of both sexes showed significantly lower body weight compared to VEH treated mice during the first 2 days of treatment. This effect normalized by the final THC treatment but reemerged during abstinence (Fig. 3I–J).

### Altered reward seeking and conditioned cue discrimination during chronic THC abstinence

**Fig. 4:**
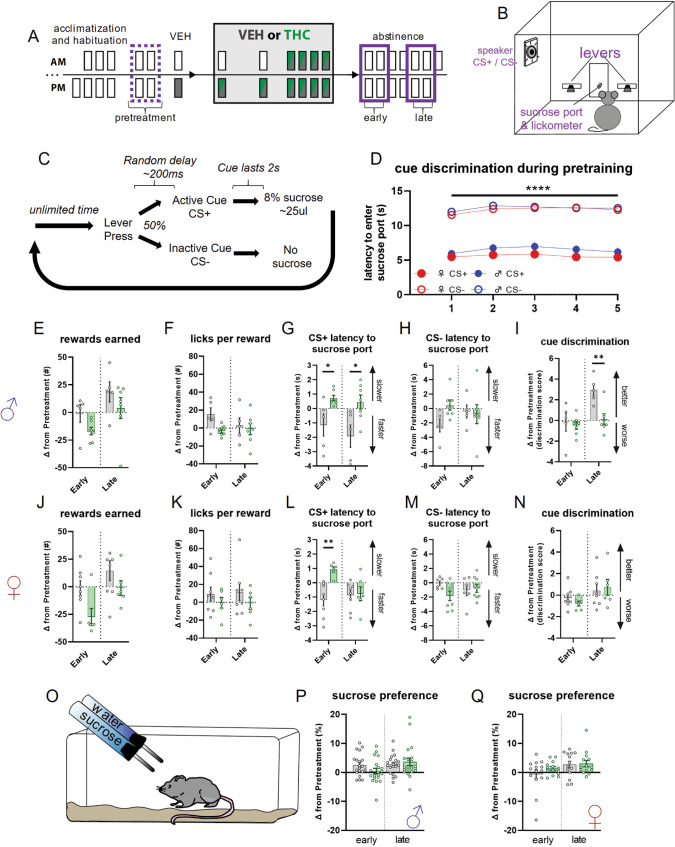
Changes in reward seeking behaviors during early and late abstinence from chronic THC treatment in male and female mice. **A** Timeline illustrating treatment regimen and epochs contributing to analysis. Dotted purple line indicates average of two pretreatment epochs that average of days 1 and 2 of early and days 5 and 6 of late abstinence are compared. **B** Cartoon illustrating operant chamber for reward seeking and conditioned auditory cue presentation. **C** Diagram showing operant sucrose seeking procedure with cue discrimination. **D** Cue discrimination during last five pretraining sessions as measured by latency to enter reward port after cue onset. There is a significant effect of cue (2_cue_ × 5_session_ RM-ANOVA; F_cue_(1,45) = 182.9; *p* = <0.0001). CS + = cue predicting reward; CS− = cue predicting absence of reward; ♂ = male; ♀ = female. **E**–**I** Changes in behavioral metrics related to reward seeking and cue discrimination in male mice. There is a significant treatment effect for change in latency to approach sucrose port after CS + (**G**; F_treatment_(1,10) = 8.616; *p* = 0.0149) with post-hoc tests finding group differences during Early (p* = 0.0261) and Late (p* = 0.0131) abstinence, and significant treatment effects in cue discrimination (I; F_treatment_(1,10) = 5.830; *p* = 0.0364) with post-hoc tests finding group differences during Late abstinence (p** = 0.0045). Non-significant effects – (E) F_treatment_(1,10) = 2.516; *p* = 0.1438. **F** F_epoch×treatment_(1,10) = 3.883; *p* = 0.0771. **H** F_epoch×treatment_(1,10) = 4.201; *p* = 0.0675. **J**–**N** Same as **E**-**I** but for female mice. There is a significant treatment effect for change in latency to approach sucrose port after CS + (**L**; F_treatment_(1,12) = 5.787; *p* = 0.0332) with post-hoc tests finding group differences during Early abstinence (p** = 0.0014). Non-significant effects – (**J**) F_treatment_(1,12) = 4.161; *p* = 0.0640. **K** F_treatment_(1,12) = 1.837; *p* = 0.1879. **M** F_epoch×treatment_(1,12) = 3.110; *p* = 0.1032. **N** F_epoch×treatment_(1,12) = 1.237; *p* = 0.2879. **O** Cartoon depicting sucrose preference test. **P**, **Q** Change in sucrose preference score during Early and Late abstinence in male (**P**) and female (**Q**) mice. No significant effects were detected. **P** F_epoch×treatment_(1,38) = 3.451; *p* = 0.0710. **Q** F_epoch×treatment_(1,26) = 1.085; *p* = 0.3071.

We hypothesized that various aspects of reward seeking and consumption would be altered during THC abstinence (Fig. 4A), consistent with observed alterations in striatal DA release and sleep disturbances. We examined these behaviors by training mice to respond on levers that led to the presentation of auditory stimuli signaling whether-or-not sucrose-solution reward would be delivered (Fig. 4B, C). Over five pretraining sessions, all mice displayed increased latency to enter the sucrose port upon lever press-contingent presentation of an auditory stimulus signaling no reward (CS−), while maintaining relatively short latency when presented with the stimulus signaling the reward (CS+) (Fig. 4D) indicating that the mice learned to discriminate CS+ from CS−.

Comparing behavior in male mice during pretreatment and following chronic THC or VEH treatment indicated no significant differences in rewards earned (Fig. 4E). THC treatment did not alter sucrose consumption behavior (i.e., ‘licks’) upon reward delivery (Fig. 4F). The most striking effect was the behavioral response to reward predictive cues, where THC treated male mice were slower to approach the sucrose reward port after a lever press indicating reward availability, i.e., CS+ latency (Fig. 4G), in both early and late abstinence. This effect was not present for non-rewarded (i.e., CS−) lever press latency (Fig. 4H). Interestingly, THC treated male mice also differed from VEH treated male mice in their change in cue discrimination during late abstinence (Fig. 4I). Here, the VEH mice increased the ratio of CS− latency to CS+ latency, while the THC mice showed ratios similar to pretreatment and early abstinence levels, indicating THC treatment attenuated the enhanced discrimination between the cues that VEH treated mice experience during late abstinence. Like male mice, THC treated female mice showed no significant differences in rewards earned (Fig. 4J), or reward consummatory behaviors (Fig. 4K), and had slower CS+ latency during early abstinence (Fig. 4L) with no difference in CS− latency (Fig. 4M). Unlike males, no effect on cue discrimination was observed in female mice for either abstinence epoch (Fig. 4N).

To examine whether differences in reward seeking behaviors were due to altered hedonic characteristics of sucrose reward, we tested mice in a sucrose preference test (Fig. 4O). No differences in sucrose preference during early or late abstinence were observed for THC treated mice of either sex compared to VEH treated mice (Fig. 4P, Q).

### Irritability measurements and circulating stress hormones

#### Altered irritability behaviors in male mice during THC abstinence

**Fig. 5:**
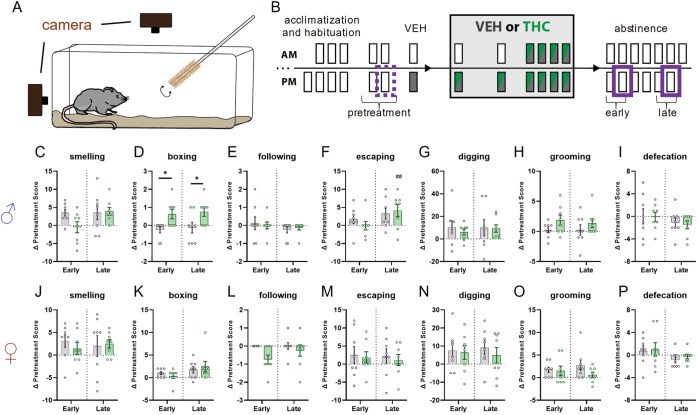
Effects of chronic THC administration on irritability-related behaviors during Early and Late abstinence in male and female mice. **A** Cartoon illustrating bottle brush test procedure for measuring irritability-related responses. **B** Timeline illustrating treatment regimen and epochs contributing to analysis. Dotted purple line indicates pretreatment session that Early and Late abstinence sessions are compared. **C**–**I** Change in irritability-related behavioral metrics during Early and Late abstinence in male (♂) mice. Significant effect of treatment was detected for boxing behavior (**D** F_treatment_(1,14) = 12.47; *p* = 0.0015) with post-hoc tests finding significant group differences in Early and Late abstinence (p* = 0.0187). Significant effect of epoch was detected for escaping behavior (**F** F_epoch_(1,14) = 8.211; *p* = 0.0125) with post-hoc tests finding within group difference in THC treated mice (*p*^##^ = 0.0096). Non-significant effects – (**C**) F_epoch×treatment_(1,14) = 2.541; *p* = 0.1333. **E** F_epoch×treatment_(1,14) = 0.7368; *p* = 0.4051. **G** F_epoch×treatment_(1,14) = 0.3984; *p* = 0.5381. **H** F_treatment_(1,28) = 3.478; *p* = 0.0727. **I** F_epoch×treatment_(1,14) = 0.0596; *p* = 0.8107. **J**–**P** Same metrics as above but for female (♀) mice. No significant effects were detected. **J** F_epoch×treatment_(1,14) = 1.246; *p* = 0.2830. **K** F_epoch×treatment_(1,28) = 0.9020; *p* = 0.3504. **L** F_treatment_(1,14) = 3.500; *p* = 0.0824. **M** F_treatment_(1,14) = 0.0392; *p* = 0.8459. **N** F_epoch×treatment_(1,14) = 0.3751; *p* = 0.5500. **O** F_epoch×treatment_(1,14) = 0.2166; *p* = 0.1632. **P** F_treatment_(1,14) = 0.0777; *p* = 0.7845.

Clinical evidence suggests some cannabis users display enhanced aggression and irritability during abstinence [13, 20, 61]. We used the bottle brush test [62, 63] (Fig. 5A) to assess changes in irritability-related behaviors during abstinence in chronic THC vs VEH treated mice (Fig. 5B).

We scored metrics related to aggressive (smelling, biting, boxing, following, exploring, tail rattling) and defensive/stress-induced behaviors (escaping, digging, jumping, climbing, defecating, vocalizing, grooming) (Fig. 5C–P). As these behaviors may be correlated within these categories, i.e., aggressive or defensive/stress-induced, we first calculated correlation matrices for each pair of behaviors for each condition (epoch, sex, and treatment). Since few significant correlations were detected (Supplementary Table 1) we report all behaviors independently. Male THC treated mice showed enhanced boxing behavior during both early and late abstinence (Fig. 5D). In addition, male THC but not VEH treated mice had a greater change in escape behaviors in late compared to early abstinence (Fig. 5F). No significant changes in irritability behaviors were detected in female mice. Biting, exploring, tail rattling, jumping, climbing, and vocalizing were scored, but were scarce or absent in most mice, so were not further analyzed.

#### THC withdrawal symptoms are not likely driven by changes in stress as measured by circulating corticosterone

Stress is known to have profound effects on sleep and behavior [64]. We assessed whether the THC treatment itself acted as a stressor in a manner that might alter sleep and wake behaviors during abstinence. We measured circulating plasma corticosterone (CORT) as an index of perceived stress and assessed changes relative to pretreatment CORT during early and late abstinence (Supplementary Fig. 8A). While THC treated male mice displayed increased CORT during early abstinence compared to VEH treated mice (Supplementary Fig. 8B), this increase (27.26 ng ± 13.17 ng; mean ± SEM) was an order of magnitude smaller than CORT increases from chronic mild stress [65]. Changes in CORT did not differ between THC treated and VEH treated female mice (Supplementary Fig. 8C).

## Discussion

### Summary

We examined how spontaneous withdrawal from chronic THC administered to naïve mice drives changes in striatal DA release, sleep disruptions, and behavioral maladaptation. Overall, our experiments show that mice can be used to study translatable THC withdrawal symptoms and neural mechanisms that might drive these symptoms. Of particular interest are apparent sex differences in nearly all metrics during spontaneous THC withdrawal. These studies open the door for further investigation into the neurophysiological changes mediating CWS and potential therapeutic interventions to treat CUD/CWS. Key findings and implications are discussed below.

### Striatal DA release and THC abstinence

Striatal DA has well established roles in drug dependence [52], including CUD [51, 66] and DA dysregulation following chronic cannabis use, is a known factor in the manifestation of CWS [67]. The implications of differing DA release in males and females during abstinence after acute THC are unclear. Perhaps the increased DA release in males makes them susceptible to enhanced sensitivity to salient or rewarding stimuli after their first drug experience, while females are resistant to such changes. Future studies are required to test whether the increased DA release in males contributes to behavioral and sleep alterations following chronic THC administration.

More pertinent to CUD/CWS are the different observations in males and females regarding DA release during abstinence after chronic THC treatment. In early abstinence, female mice had far greater DA release across the striatum, while males showed no such effects. However, while in females these measurements returned to control level in late abstinence, the relative lack of change in DA release in males may result in susceptibility to changes in reward-associated stimuli. These findings may be related to effects of altered dopaminergic transmission in disrupted cognitive function due to dysregulation in signal to noise processing [68]. Again, further investigation into the role of altered DA release in wake behaviors and sleep is needed.

Previous studies have mainly examined effects of chronic cannabis or THC on firing of ventral tegmental area DA neurons that project to striatum, with less information about changes during abstinence. Furthermore, it is unclear how these firing changes relate to alterations in striatal DA release, as that has not been measured in past studies. Diana and coworkers found that rat midbrain DA single-unit activity was reduced 24 h after the last THC injection using a treatment regimen similar to the one we used [69]. Based on this finding one might not expect the increased striatal DA release that we observed during abstinence. A possible explanation for this contradiction is the recent evidence that striatal DA activity does not necessarily reflect midbrain DA neuron activity [70], and this dissociation can be explained by intrastriatal microcircuitry. Indeed, the endocannabinoid (eCB) system can modulate striatal DA release independent of postsynaptic action potentials [71], suggesting prolonged alterations in striatal eCB action due to THC administration as a potential contributor to increased DA release. Imaging studies in human cannabis users abstaining for 1–7 days found a reduction in stimulant-induced DA response [67], which also contrasts with our findings of increased DA release at similar timepoints during abstinence. Technical differences can potentially reconcile these divergent findings, as stimulant-mediated increases involve altered DA transporter function while electrical stimulation induces DA release directly via DA afferent stimulation and indirectly via cholinergic interneuron activation [72]. Examining the role of cholinergic drive on DA release may thus also indicate mechanisms underlying THC actions. Likewise, our data were collected postmortem in brain slice preparations taken at precise timepoints during abstinence. Future in-vivo studies using genetically coded DA sensors [73] can also shed light on the relationship between striatal DA activity and precise timepoints during abstinence from THC.

### A mouse model for sleep disruption during THC withdrawal

Treating sleep pathologies is often given as a reason for THC use both recreationally and medically [74, 75]. Indeed, many individuals list poor sleep as a major factor leading to their relapse to cannabis use [16], and those subjects showing sleep disruption, poor sleep quality in particular, relapse more readily [26, 27, 76, 77]. However, sleep disruption is one of the most consistent and problematic aspects of cannabis withdrawal, with altered sleep observed in the majority of regular users who attempt to quit [16, 21].

The observed effects on sleep, especially in male mice, are generally consistent with our hypothesis that chronic THC administration would cause sleep disruption that mirrors the transient changes observed in the clinical setting, and are consistent with a study using the synthetic CB1 agonist AM2389 [78] that was published while the present study was under consideration for publication. The observations that acute THC treatment enhanced NREM while REM sleep was fragmented in male mice during abstinence are consistent with previous reports of single THC or cannabis exposures in animal models [79–81] and humans [32, 82–86]. The finding that THC tolerance following chronic treatment diminishes the drug’s effect on sleep is also consistent with past findings in animals [80, 81, 87–89] and regular human cannabis users [34, 82, 85, 90–92]. We also found that male mice experience disruptions during THC abstinence similar to human cannabis users [35, 36, 59, 93, 94] while female mice appear more resilient to the overall effects of spontaneous THC withdrawal on sleep. The absence of significant differences in female mice in our study was surprising given that female cannabis users report sleep difficulty during cannabis abstinence [95]. However, in clinical polysomnographic studies measuring sleep during cannabis abstinence the participants are primarily male, making comparison to our findings in female mice difficult. Future clinical studies should be properly powered to examine sleep physiology in female cannabis abstainers. Additionally, if female cannabis users are indeed more resilient to sleep changes as measured by polysomnography, there are other psychological manifestations occurring during cannabis or THC abstinence that may drive self-report of poor sleep quality despite no profound changes in sleep physiology. We believe overall our study provides a valuable back-translational model to investigate the neural mechanisms mediating sleep disruption during cannabis withdrawal.

### Mouse as a back-translational model for CWS during wake-behaviors

Past research using mice to study spontaneous CWS primarily report negative findings for behaviors beyond typical somatic symptoms and cannabis tetrad metrics [3]. This is potentially confounded by typical behavioral paradigms used in preclinical settings that may not adequately probe the most pronounced human CWS. We focused our behavioral tasks to measure changes in behaviors that directly map to some of the strongest CWS; (1) reduced food and water intake, (2) restlessness (i.e., heightened locomotion), (3) amotivation, (4) attention deficits, and (5) irritability.

We observed profound hypophagia, hypodipsia, and reduced locomotion after the first THC injection, and our chronic THC treatment clearly induces behavioral tolerance to the effects of the drug as evidenced by the normalization of these measures to control levels by the final THC injection. However, the lack of change in these metrics in abstinence following chronic THC exposure suggests home cage intake and locomotion may not serve as an appropriate back-translational model for human CWS. Perhaps this paradigm does not recapitulate other environmental and cognitive factors that manifest in the human population, e.g., additional life stressors, or situations that when compounded with cannabis abstinence affect food and water intake. Nonetheless, we observed significant differences between treatment groups in body weight as early as day 2 of abstinence. This finding is particularly intriguing as it suggests chronic THC treatment alters metabolic function that may contribute to alterations in body weight despite no differences in intake or locomotion – a notion ripe for future investigation.

Our observation that both male and female mice did not differ in rewards earned indicates that CWS in mice does not involve strong changes in motivation or drive. However, the slower retrieval of reward in THC treated mice of both sexes may indicate decreased motivation. Responses to the CS+ and CS− cues that followed operant responding provide insight into attention during this goal-directed task. In particular, poorer cue discrimination in male mice treated with THC in late abstinence indicates a deficit in attention or focus that emerges later in abstinence. While this operant responding paradigm provided evidence of impairment in several cognitive processes related to goal-directed action during chronic THC abstinence, future studies should directly target specific aspects of reward seeking behaviors and attention processes in an effort to decipher the neural mechanisms mediating these specific disruptions.

Increased irritability is prevalent during cannabis abstinence in humans [24, 96]. We were surprised to find that relatively few irritability-like behaviors were altered in our bottle brush test assay. Nonetheless, male mice showed enhancement of some irritability-like metrics, i.e., boxing and escape behaviors. These behaviors are interesting in that they demark disparate types of irritability behaviors – aggressive and defensive, respectively, indicating that male mice experience alterations in irritability behavior in general, though these alterations manifest in somewhat specific behavioral outputs in the bottle brush test.

### Conclusions and caveats

Technical limitations to our studies, primarily that metrics were gathered from separate cohorts of animals, make it difficult to draw conclusions about correlations between various behaviors and, in particular, DA voltammetry measurements. Nonetheless, a general observation is that female mice appear more resilient to effects of chronic THC treatment on sleep and maladaptive behaviors during abstinence. A speculative explanation is the elevation in striatal DA release we observe in female mice during early abstinence may play a protective role in attenuating withdrawal symptoms, and the clearest indicator of this effect in our studies is in the relationship between lack of sleep disruption and striatal DA release in early abstinence in female mice. Striatal DA is known to play a role in sleep-state architecture [46], so further studies are needed to investigate the causal role of enhanced striatal DA release observed in female mice in alleviating sleep disruption during early chronic THC abstinence.

Beyond the changes in striatal DA release we observed in female mice, we can speculate about other physiological mechanisms that may contribute to the lack of abstinence effects in female sleep or wake behavior in our spontaneous THC withdrawal assays. In several of our assays, female data appear more variable than males. This could be due to influences of sex-hormone cycling, which are known to influence sleep [97] and motivated behaviors [98]. Likewise, THC is lipophilic and can be stored in adipose tissue [99]. In general, male rodents have larger fat stores than females which could influence behaviors during abstinence, due to transient THC release from this tissue reserve [100]. However, for several of our assays, we use an average of 2 days of measurements which may mitigate any such effects. Another factor might be innate differences in the eCB system between males and females [101, 102]. We hypothesize, and have shown here for sleep measurements, that neurophysiological changes that drive tolerance are critical for the manifestations of CWS, and as such females may express these changes differently than males, despite both sexes showing evidence of tolerance in our treatment procedure.

Additionally, it is clear that the light cycle plays a role in CWS manifestation in mice, particularly with respect to sleep. Several of our assays were conducted at one timepoint (either during dark phase or light phase) and further studies should be done to address the role of circadian rhythms in the strength of CWS in mice.

Our findings provide models of significant and translatable CWS in rodents using a protocol that does not require antagonist-precipitated withdrawal. Note that withdrawal from misused drugs is a complex psychophysiological phenomenon, and our aim here is to present preclinical assays that have both successfully and unsuccessfully modeled distinct CWS. Future studies based on the treatment protocol and timepoints used in our study can unravel the likely complex neural mechanisms that drive sleep disruption and behavioral maladaptation prevalent in CWS – crucial first steps towards therapeutics to combat CUD.

## Supplementary information

Supplementary Information
