# Supplementary material for: Changes in striatal dopamine release, sleep, and behavior during spontaneous Δ-9-tetrahydrocannabinol abstinence in male and female mice
Source: Neuropsychopharmacology. 2022 Apr 27;47(8):1537–49. doi: 10.1038/s41386-022-01326-0 (PMC9205922; doi:10.1038/s41386-022-01326-0)
Supplement: Supplementary file 1 — Supplementary Information [file 41386_2022_1326_MOESM1_ESM.docx]

# Supplemental Information

**Changes in striatal dopamine release, sleep, and behavior during spontaneous Δ-9-tetrahydrocannabinol abstinence in male and female mice**

Andrew J. Kesner^1,2^, Yolanda Mateo^1^, Karina P. Abrahao^3^, Stephanie Ramos-Maciel^1^, Matthew J. Pava^4^, Alexa L. Gracias^1^, Riley T. Paulsen^1^, Hartley B. Carlson^1^, and David M. Lovinger^1^

^1^National Institute on Alcohol Abuse and Alcoholism, Intramural Research Program, NIH, Bethesda, MD, USA.

^2^Center on Compulsive Behaviors, Intramural Research Program, NIH, Bethesda, MD, USA.

^3^Departamento de Psicobiologia, Universidade Federal de São Paulo, Campus São Paulo, SP, Brazil.

^4^Arlington, VA, USA.

# Supplemental Methods and Materials

## Subjects

C57Bl6/J mice were obtained from the Jackson Laboratory (Bar Harbor, ME), and housed 2 to 4 per cage. Initially, mice weighed 25 to 30 g, and their body weight did not change substantially throughout the study. Upon arrival to the animal colony, all mice were maintained on a 12:12 h light:dark cycle with lights turning on at 06:30 EST and off at 18:30 EST. Throughout all parts of the study, except during hour long operant responding experiments, subjects were provided with ad libitum access to food and water and housed in an environment maintained at 22.2 °C and 50% humidity. The experimenter conducting experiments where data was collected was blind to experimental treatment groups, except for measurement of weights during chronic treatment administration.

## Sleep recordings

### Surgical preparation for sleep recordings

Surgeries were performed on subjects anesthetized with isoflurane. Custom electrode implants were prepared as previously described [[1](#_ENREF_1)]. Stereotaxic surgery was performed to implant electrodes over medial frontal cortex (AP: +1.50, ML: ±1.00) and parietal cortex (AP: −2.50, ML: ±2.00). A ground/reference electrode was also implanted over the cerebellum (AP: −5.7, ML: ±1.7). Electrodes consisted of stainless-steel screws (AMS90/1P-25; Antrin Miniature Specialties Inc., Fallbrook, CA) placed in contact with the brain surface and wrapped with Teflon-insulated fine stainless-steel wires (Cat # 91400; A-M Systems Inc., Sequm, WA), that in turn connected to a headcap (MS363 & E363-0; Plastics One, Roanoke, VA) for later connection with recording tethers (363-363; Plastics One, Roanoke, VA). A 1.25mm stainless steel suture pad also connected to the headcap was inserted beneath the nuchal muscle before the entire headcap was fixed to the skull using standard cold-cure dental acrylic. Post-operative pain and discomfort were mitigated by administering ketoprofen (5 mg/kg i.p.) analgesic solution immediately after the surgery was completed and once daily for the next two days. Following surgery, subjects were allowed to recover for two weeks before being tethered to recording equipment.

### Recording environment.

After the recovery period mice were brought to an isolated room dedicated to 24-hour, long term, mouse sleep recording experiments. Subjects were placed individually into recording cages (custom fabricated, 4 L polycarbonate buckets, Cambro RFSCW4135, Webstaurant Store, Lancaster, PA) and tethered to commutators (SL6C/ SB; Plastics One, Roanoke, VA). These cages were placed into electromagnetically shielded chambers that provided sound and light attenuation (SAE chambers; 5 cages/chamber; CT-ENV-018MD-EMS-X1, MED Associates, Fairfax, VT), with five individual cages present in each chamber such that mice could see, smell, and hear one another. The chambers were outfitted with small fans which continuously circulated room air and provided stable background noise within the recording chambers, and white LED light strips (#10434, General Electric, Fairfield, CT) controlled by a timer synchronized with ambient room lighting. Standard food pellets (BioServe; Cat no. NIH31) were placed on the cage bedding and access to water was provided via glass tubes (#9019, Bio-serve, Frenchtown, NJ). White noise generators were continuously running in the recording room itself to further mask outside noise from disturbing mice.

### Polysomnographic (EEG/EMG) recording.

Recordings of polysomnographic signals (EEG/EMG) were obtained from the chronically tethered mice over 23hr periods. During the intervening 1hr, data acquisition was halted to check on the animals to ensure ad libitum food and water were available and perform injections of THC or vehicle when appropriate. EEG/EMG signals were amplified 1000x (20x HST/16V-G20 head-stage followed by 50x wide-band PBX, Plexon, Dallas, TX) and digitized at 1 kHz (PCI-6071E, National Instruments, Austin, Tx). Data collection used a standard PC computer (Optiplex GX620, Dell Computers, Round Rock, Tx) running Recorder v2 software (Plexon). A 60 Hz digital notch filter was applied to attenuate line noise on all channels. EEG channels were then low-pass filtered at 120 Hz with a 2-pole Bessel filter, and EMG channels were high-pass filtered 40 Hz with a 4 pole Bessel filter. Data were saved to disk for analysis offline.

### Scoring of vigilance states and sleep architecture

#### **Constructing the state space**

EEG/EMG signals were analyzed offline to categorize epochs of wake, NREM, and REM states using software written int MATLAB and CUDA C. This methodology has been previously validated in our laboratory [[1](#_ENREF_1)] and has been used in several sleep-wake architecture analysis [[1](#_ENREF_1), [2](#_ENREF_2)]. The following analysis pipeline was conducted for each mouse for each recording session. Briefly, data were scored in 2 s epochs by clustering power spectral features in a 3-dimensional state space. The three power spectral features that made up the state space orthogonal vectors were: (1) a power spectral ratio (0.5–20 Hz/0.5–100 Hz) from the frontal EEG channel, known to separate NREM from wake and REM [[3](#_ENREF_3)]; (2) a second power spectral ratio from the occipital EEG channel to compute the prominence of theta and low alpha relative to delta power (5–10 Hz/0.5–4 Hz). This ratio has been used to help separate REM epochs which are characterized by an overall increase in the theta bandwidth [[4-6](#_ENREF_4)]; and (3) the root mean square values of the EMG power spectra. Finally, each of these state space vectors was smoothed by convolution via a 10 s Hann window, median-centered, and normalized to the maximum absolute value.

#### **Vigilance state classification.**

A three-step process was implemented to categorize vigilance state once the state space was calculated [[1](#_ENREF_1)]. First, a rough estimate of the boundaries for each state based on univariate distributions along each axis were used to produce a starting point for establishing a preliminary score. Next, a 3-dimensional product kernel estimate using a Gaussian kernel function was calculated for each cluster (i.e., wake, NREM, REM, and unassigned) and these estimates were scaled such that their maximal values were equal to the value in the corresponding peak of the overall density function. We established 99% confidence intervals from this estimate that formed inclusion criteria for the three clusters. Finally, we applied a transitional classifier to clean up unassigned points that were bounded by epochs of the same state. The scoring results for each subject were visually inspected to ensure accuracy, and subjects with excessive noise in the EEG/EMG signals that interfered with scoring were eliminated from further analyses.

Once we obtained reliable vigilance state classification for each 2 s epoch, the data were then binned into 3 hr time windows and the percent time spent in either REM, NREM, or wake were calculated along with the total number and duration of each bout of these states. The final 3 hr bin (ZT 9:00 thru 11:59) was excluded from analysis because this is when recordings were interrupted for animal husbandry and drug treatments. A “bout” was defined as a contiguous block of 2 s epochs assigned to the same state, and the total number of bouts is the sum of bouts over the 3 hr bin. The bout duration metric was calculated by averaging the number of 2 s epochs per bout, and then multiplying this number by 2 s (i.e., the duration of each 2s epoch).

## Behavioral testing procedures

#### Sucrose preference test

To pre-expose mice to individual housing and the two-bottle choice (sucrose preference test) procedure, mice were anesthetized and ear punched for future identification, then after a short recovery time, were separated into individual cages with a nestlet and food ad lib. Using standard home cage lids equipped for inserting two sippers/bottles side by side, we gave mice two water bottles for the first 23 hr period. Bottles were placed in the lid ~15-30min before lights off. After the first 23hrs with two water bottles, we provided mice with one bottle containing water and one containing 1% sucrose in facility water. Both bottles were weighed to compare later for obtaining amount of liquids consumed, and the side which contained sucrose and water was counterbalanced for all mice. The mice were then placed in a back room with the same lights on and off schedule and a white noise machine to mask any outside noises. The room was not entered for the duration of drinking for any drinking period. The data for this first session not analyzed because of initial side biases of mice. That is, this first session was an opportunity for mice to sample each liquid and learn that there were two different liquids. The same procedure was performed for two more 23hr periods (i.e., 2 days of pretreatment), with side assignment of sucrose and water bottles reversed each night. After finishing the pretest experiments, mice were again group housed with their original housemates (4 per cage) and were allowed to reacclimate to group housing for 36 hours. Then the mice were given the 6-day THC (10mg/kg) treatment, with 2 mice in each cage receiving THC injections and the other two mice receiving vehicle injections each day. Twenty-four hours after the final injection, the mice were again separated into individual cages and provided two bottle choice of the same sucrose and water solutions, and left in the same room, under the same conditions, for the same time periods as in the pretest. Bottles were measured every 24 hours for the next six days of abstinence. Sucrose preference was inferred by the percent sucrose consumed of total liquid volume consumed.

#### Conditioned operant sucrose seeking.

Mice were group housed, four per cage, throughout the experiment. Mice were acclimated to water restriction over several days to potentiate acquisition of operant sucrose seeking. Experiment sessions were conducted in standard operant conditioning chambers (Med Associates, ENV-307W). Each chamber was equipped with two levers, two cue lamps, a house lamp, an audio stimulus generator, a syringe pump for reward delivery, and a reward port equipped with a contact lickometer (ENV-303RMW-4.25). Operant sessions were conducted between ZT700 and ZT1000.

*First training procedure:* Both levers are available throughout the 30-minute session. A lever press on either lever resulted in a two-second auditory cue (CS+), which began after the lever-press, followed by delivery of the sucrose solution (~20 uL of 8% sucrose, 1% maltodextrin, in facility water) at the end of the cue. Additional lever-presses had no result until mice retrieved the sucrose reward as detected by breaking the photobeams in the sucrose port. Occasionally sucrose solution droplets were added to the levers to facilitate approach to the lever and operant responding. Mice had to successfully earn >21 rewards during the session before moving to the second training procedure.

Second training procedure: This procedure was identical to the first training procedure, except a second auditory cue was added to serve as a predictor of no sucrose reward (CS-). CS+ occurred after 50% of lever presses and CS- after the other 50% in a random fashion. Mice quickly learned to discriminate between CS+ and CS- and after 3 sessions were no longer water restricted.

Third training procedure: This procedure was identical to the second training procedure, but mice had access to water ad-lib in home cage. This lasted an additional 5 daily sessions, with the final two sessions comprising the data for pretreatment condition epoch.

#### Food and water intake and locomotor activity monitoring.

Mice were individually housed in Sable Systems International Promethion 3721 Mouse Cage (8.1 × 14.4 × 5.5 in). Cages were equipped with load-cells to monitor food and water intake each second (part # MM-1) and cages were surrounded by photobeams (part # - BXYZ), allowing for precise measurement of consumption and locomotor activity. Cages were lined with 1/8” Teklad corncob bedding (Catalog# 7092). Mice were given a nestlet and an enrichment device (a solid, plastic cube which while placed beneath the food hopper deterred nesting beneath the feeder). After a five-day acclimation period, mice began the treatment and abstinence procedures that were identical to sleep experiments. Mice were also weighed daily at ZT11:00 beginning just prior to vehicle treatment.

After recordings were completed, data were extracted from the Sable Systems acquisition files using their standard analysis “macros”. First a macro (“macro1”) was run on the raw data files to extract the data, and a second macro (“macro13”) was minorly edited and run on these preprocessed data to bin the data and save in comma separated (csv) format. These postprocessed csv files were then opened in Microsoft excel and further grouped or averaged into epochs of interest and then transferred to GraphPad Prism for analysis and figure plotting.

#### Bottle brush test.

The bottle brush test (BBT) was adapted from previous publications [[7-9](#_ENREF_7)]. BBT sessions were conducted between ZT1300 and ZT1500. Mice were handled by the experimenter for 5 minutes per day for 3 days prior to beginning the BBT experiment. On test days, home cages were covered with a sheet to block ambient light and then transferred to the behavioral suite. The behavioral suite was equipped with white-noise sound generators and was illuminated with red-ambient light only. The testing arena was separated from the ‘holding’ area by a door so that mice being tested and cage mates were sensory-isolated from each other. The experimenter wore a red-light headlamp. Ten min before its test session, a mouse was put in a fresh home cage with clean bedding and allowed to habituate. Next, the cage was picked up and transferred to the BBT recording area, which was a roofless, white-walled 2 ft x 2ft x 2ft enclosure, and the walls of the animal’s cage were extended by stacking on top an additional “floorless” identical cage. These two testing area features achieved, respectively, 1) separating the animal visually from the rest of the behavioral room and 2) preventing the mouse from jumping out of its test cage while the BBT was conducted.

Mice were group housed, 4 per cage, for the duration of the experiment except for separation one-by-one as described above during the BBT itself. The test itself was performed in its entirety four separate times. First on the third day of handling (pre-exposure), then again 48hrs later (pretreatment), followed by the 6-day chronic THC/vehicle treatment procedure, and then on days 2 and 6 of abstinence. Treatment groups were assigned randomly, with 2 mice from each cage receiving THC and the other 2 mice vehicle.

During the test, irritability behaviors were elicited by a rotating bottle brush (NSN 7920-00-297-1510) with brown bristles that extended ¾ inch radially for 3.5 inches lengthwise, which was attached to a ¼ inch diameter x 2 ft metal rod that allowed the experimenter to remain at a distance from the test cage. Each attack consisted of five “stages” in the following sequence: 1) rotating brush (180 degrees about 2x/sec; with brush spinning axially via twisting of the rod in the experimenter’s fingers) toward the mouse from the opposite end of the cage, 2) continuing to rotate the brush, but now against the animal’s whiskers, 3) rotating brush moving away from mouse back to opposite end of cage, 4) rotating brush at the starting position, 5) brush at starting position without rotating. Each stage was designed to elicit various aspects of murine irritability responses, and stage lasted 2 seconds, except the 5th stage that was prolonged until the mouse returned to its end of the cage or after 5 seconds. This was repeated for each animal 10 times per test, with 10-15 second intertrial intervals. Cage-mates were habituated in a staggered manner and kept outside the testing room until testing, with the order between treatment groups counterbalanced within and between cages. Bottle brushes were switched between mice to prevent responses to conspecific scents. All brushes were cleaned with ethanol and left to dry before being used for another testing day.

BBT sessions were recorded using Bonsai RX software (10.3389/fninf.2015.00007) to record synchronous video and audio. Video (30 fps) was recorded using two standard USB web cameras without infrared filters (E.L.P.; Ailipu Technology Co., China) mounted above and to the side of the testing cage, and ultrasonic audio (384khz) was recorded from a high-frequency microphone (M500; Pettersson Elektronik AB, Sweden) mounted 18 inches above the testing cage. Behaviors were scored manually via top and side recorded video. Overall scores for the various behaviors are results from two independent scorers. When scores for a particular behavior diverged between scorers, the scorers met and resolved the discrepancy. Generally, scores were in agreement. Responses were designated as aggressive or defensive as described by [[9](#_ENREF_9)]. Responses scored were smelling, biting, boxing, following, exploring, tail rattling, escaping, digging, jumping, climbing, defecating, vocalizing, and grooming. Only one scorer reviewed audio data since there was a clear lack of ultrasonic vocalizations in the recordings. The same recording setup was used to record vocalizations during male and female interactions resulting in a positive control for ultrasonic vocalization.

## Plasma corticosterone collection

Pretreatment samples were collected the day before the initial 10mg/kg THC injection of the treatment paradigm, while early and late samples were collected two days and six days after the final treatment, respectively. Mice were restrained using a Tailveiner Restrainer (TV-150) such that the mouse was placed into the device tail-first. The tail was gently rubbed with a hand warmer for 5 seconds to increase blood flow. A carbon steel scalpel blade (size 11, Integra) was used to snip the lateral tail vein and blood was collected using a heparinized Micro-Hematocrit capillary tube (Fisher Scientific, PA). Pressure was applied to the snip site to stop excessive bleeding and the mouse was placed in a clean new cage within the room where sample collection occurred. Cage mates were kept outside the blood sampling room until blood collection. Sampling took no longer than 2 minutes per mouse. The capillary tubes were spun in a DM1424 Hematocrit Centrifuge (SCILOGEX, LLC, CT, USA) to separate red blood cell and plasma components. 15-30 uL of plasma was transferred into an Eppendorf tube and stored in a -20 C freezer until further analysis.

## Statistical analysis

For sleep studies we analyzed the data by fitting a mixed model as implemented in GraphPad Prism 9.0. This mixed model uses a compound symmetry covariance matrix and is fit using Restricted Maximum Likelihood (REML). In the absence of missing values, this method gives the same P values and multiple comparisons tests as repeated measures ANOVA. In the presence of missing values, the results can be interpreted like a repeated measures ANOVA. For these data a three-way (2_photoperiod_ x 2_epoch_ x 2_treatment_) repeated measures mixed-effects model was used, allowing us to analyze the data set despite missing values for some subjects on some recording sessions. These occurred at random due to scenarios such as, mice breaking recording tethers, moisture accumulation from water during a recording session leading to static electricity artifacts/noisy recordings, and headcaps or recording tethers becoming detached. For consistency, the same analysis was used for 24hr intake and locomotion behavior recordings, and all other behavioral studies used a two-way 2_epoch_ x 2_treatment_ repeated measures mixed-effects model. When significant (p<0.05) main and interaction effects relating to treatment were detected, we followed up with Holm-Šídák's multiple comparisons post-hoc test to compare means of metrics differing by only one factor. The non-significant effects presented in the main text figure legends were the conditions related to treatment that had the lowest p-value. Since photoperiod has clear effects on sleep regardless of treatments or epochs, we only report significant post-hoc tests comparing treatment effects and within-subjects effects related to epoch. For voltammetry experiments a two-way ANOVA were used for statistical comparisons between groups.

# Supplemental Figures

## Supplemental Figure 1


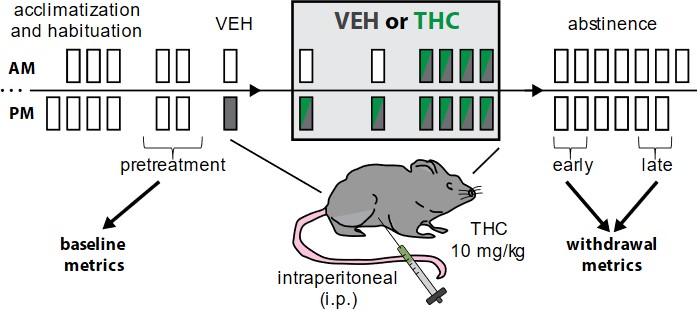


### General timeline used to model chronic cannabis/THC use in mice.

Time points allow comparison of pretreatment epochs to early and late abstinence, and initial vehicle injection to ‘first’ and ‘last’ THC or VEH injections during treatment epoch.

## Supplemental Figure 2

##
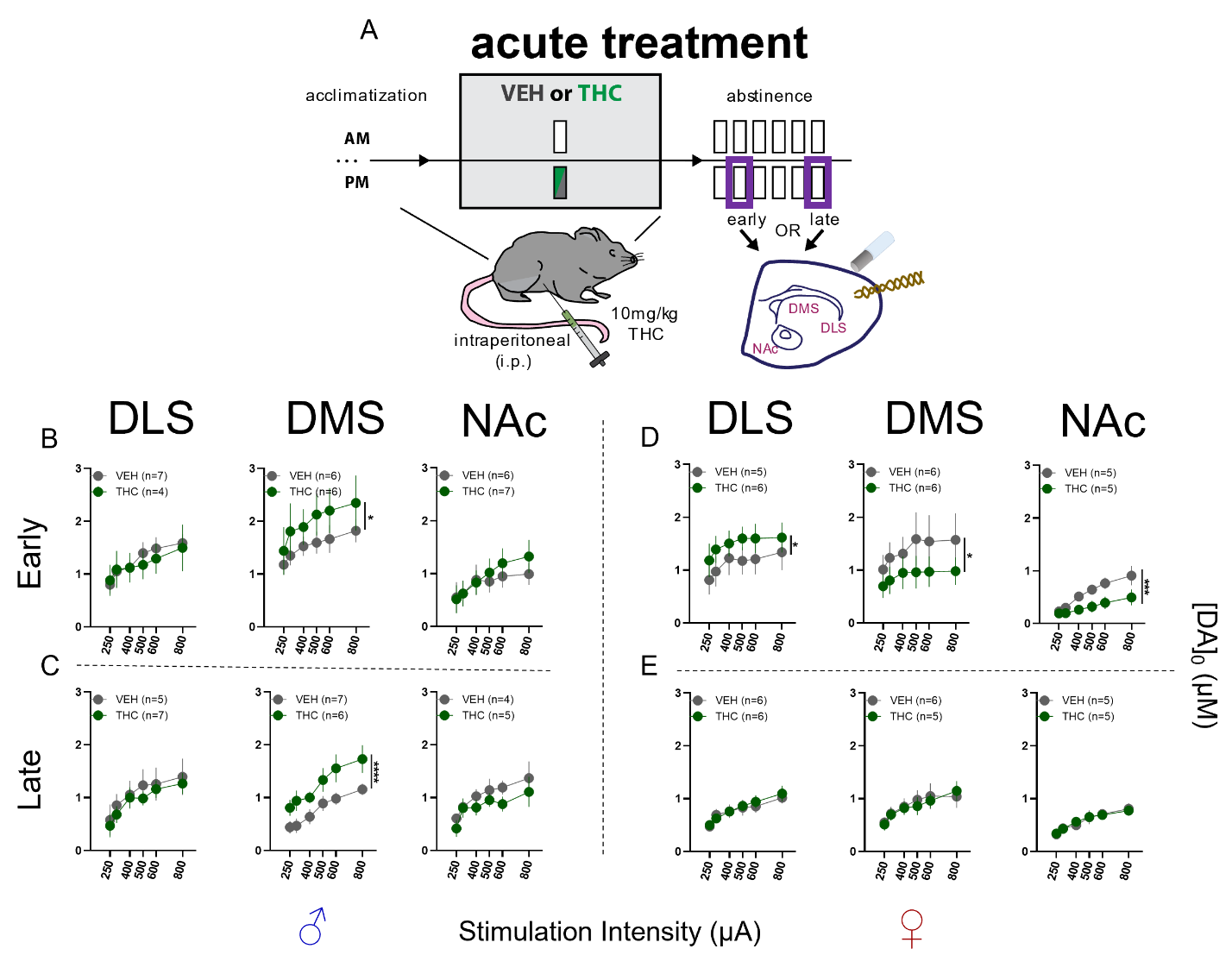


### Effects of acute THC treatment on striatal DA release in acute brain slices using FSCV.

1. Schematic illustrating acute treatment and time points of FSCV recordings in striatal subregions where a carbon fiber recording electrode and stimulating electrode were placed to record electrical stimulation-elicited DA release.
2. DA release in males during early abstinence following acute treatment. No differences detected in DLS or NAc. In DMS THC treated mice had elevated DA release (F_treatment_(1,60) = 4.866; p = 0.0312)
3. DA release in males during late abstinence following acute treatment. No differences detected in DLS or NAc. In DMS THC treated mice had elevated DA release (F_treatment_(1,59) = 21.33; p = <0.0001)
4. DA release in females during early abstinence following acute treatment. In DLS THC treated mice had elevated DA release (F_treatment_(1,54) = 4.726, p = 0.0341), while DA release was reduced in DMS (F_treatment_ (1,60) = 5.669; p = 0.02) and NAc (F_treatment_(1,48) = 15.87; p = 0.0002).
5. DA release in females during late abstinence following acute treatment. No differences detected in DLS, DMS, or NAc.

## Supplemental Figure 3


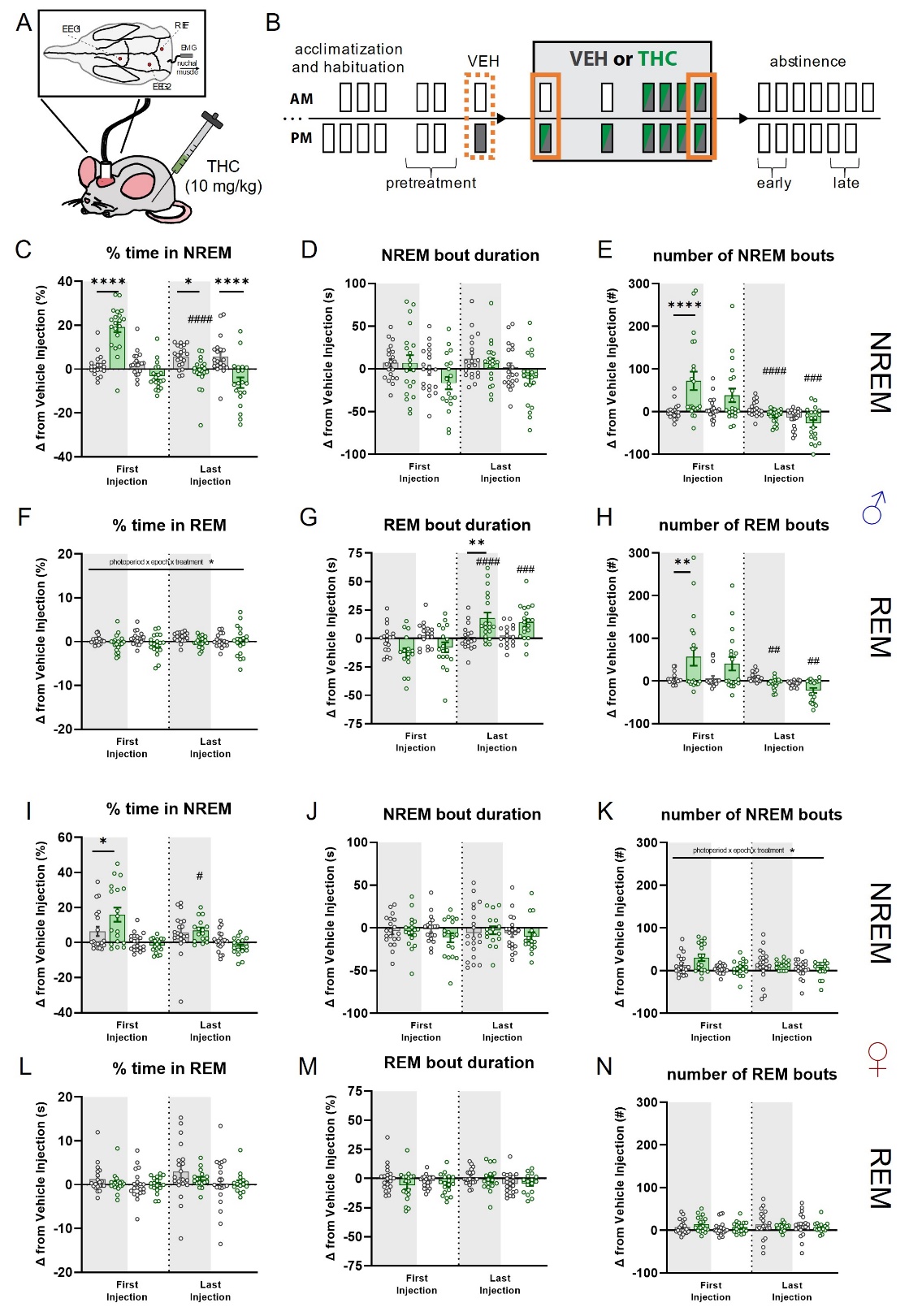


### Effects of acute and chronic THC administration on sleep in male and female mice.

1. Cartoon illustrating i.p. administration of 10mg/kg THC in chronically tethered mice implanted with two EEG, one EMG, and one reference electrodes and their relative placements on the skull / nuchal tissue.
2. Timeline illustrating treatment regimen and epochs contributing to analysis. Dotted orange line indicates vehicle treatment epoch that acute and chronic administration epochs are compared.
3. Comparison between effects of THC or VEH on changes from vehicle injection in percent time spent in NREM sleep in male mice. There is a significant interaction (F_epoch x treatment_(1,38) = 66.18; p = <0.0001), with post-hoc tests finding differences between-groups during First Injection-LOFF (p**** = <0.0001), Last Injection-LOFF (p* = 0.0346) and LON (p**** = <0.0001). The THC group had significant within-group difference between First and Last Injection LOFF (p^####^ = <0.0001).
4. Comparison between effects of THC or VEH on changes from vehicle injection in NREM bout duration in male mice. No significant effects were detected.
5. Comparison between effects of THC or VEH on changes from vehicle injection in number of NREM bouts in male mice. There is a significant interaction (F_epoch x treatment_(1,38) = 11.68; p = 0.0015), with post-hoc tests finding differences between-groups during First Injection-LOFF (p**** = <0.0001). The THC group had significant within-group difference between First and Last Injection LOFF (p^####^ = <0.0001) and LON (p^###^ = 0.0001).
6. Comparison between effects of THC or VEH on changes from vehicle injection in percent time spent in REM sleep in male mice. There is a significant interaction (F_photoperiod x epoch x treatment_(1,30) = 4.573; p = 0.0407).
7. Comparison between effects of THC or VEH on changes from vehicle injection in REM bout duration in male mice. There is a significant interaction (F_epoch x treatment_(1,36) = 27.37; p = <0.0001), with post-hoc tests finding differences between- groups during Last Injection-LOFF (p** = 0.0112). The THC group had significant within-group difference between First and Last Injection of LOFF (p^####^ = <0.0001) and LON (p^###^ = 0.0002).
8. Comparison between effects of THC or VEH on changes from vehicle injection in number of REM bouts in male mice. There is a significant interaction (F_epoch x treatment_(1,36) = 9.043; p = 0.0048), with post-hoc tests finding differences between-groups during First Injection-LOFF (p** = 0.0051). The THC group had significant within-group difference between First and Last Injection LOFF (p^##^ = 0.0011) and LON (p^##^ = 0.0013).
9. Comparison between effects of THC or VEH on changes from vehicle injection in percent time spent in NREM sleep in female mice. There is a significant interaction (F_photoperiod x treatment_(1,38) = 5.881; p = <0.0202), with post-hoc tests finding differences between-groups during First Injection-LOFF (p8 = 0.0215). The THC group had significant within-group difference between First and Last Injection LOFF (p^#^ = 0.0422).
10. Comparison between effects of THC or VEH on changes from vehicle injection in NREM bout duration in female mice. No significant effects were detected.
11. Comparison between effects of THC or VEH on changes from vehicle injection in number of NREM bouts in female mice. There is a significant interaction (F_photoperiod x epoch x treatment_ (1,25) = 6.629; p = <0.0163).
12. - (N) Comparison between effects of THC or VEH on changes from vehicle injection in percent time in REM (L), bout duration (M), and number of bouts (N) in female mice. No significant effects were detected.

## Supplemental Figure 4


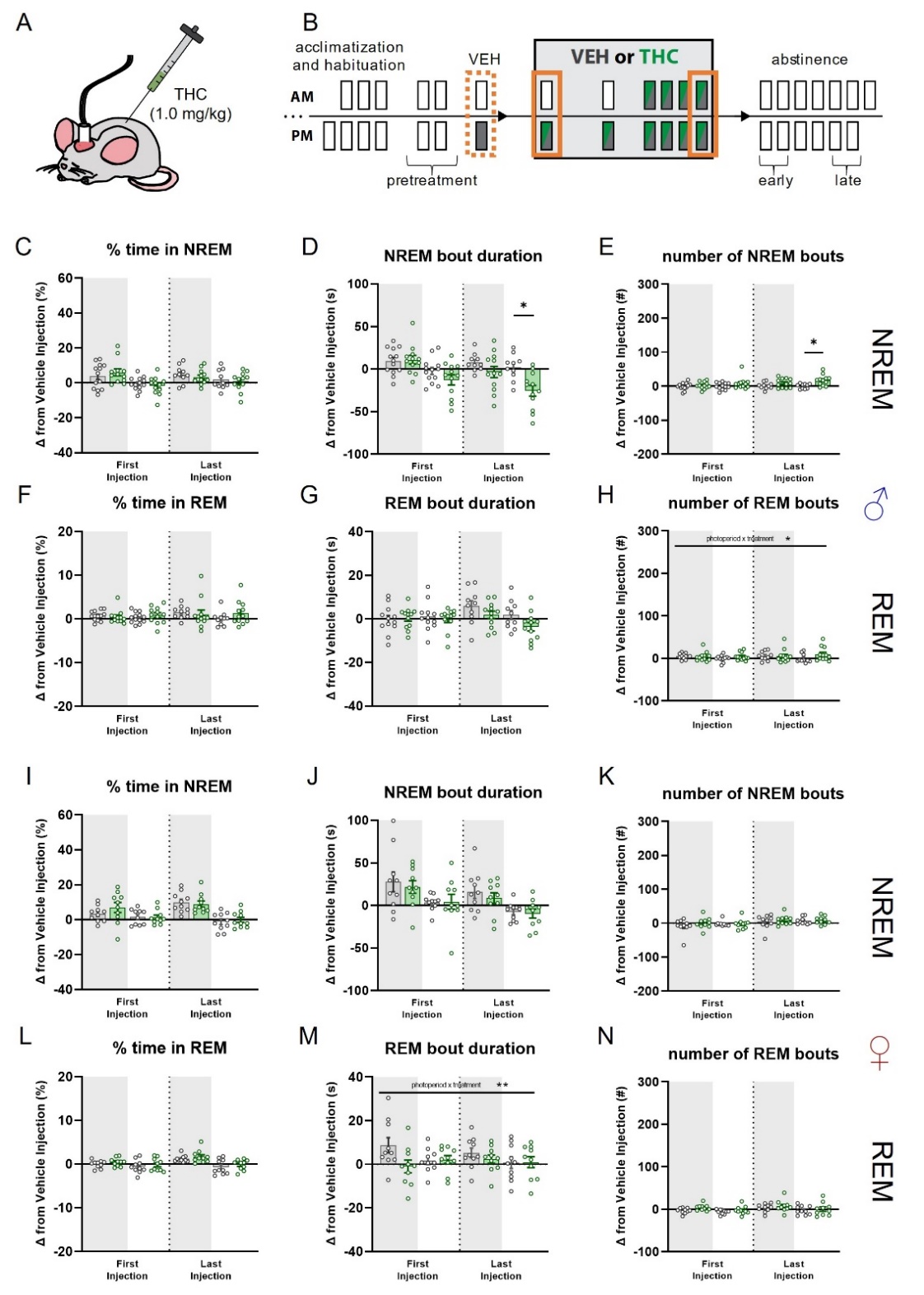


### Effects of acute and chronic low dose THC administration on sleep in male and female mice.

1. Cartoon illustrating i.p. administration of lower dose, 1.0mg/kg THC.
2. Timeline illustrating treatment regimen and epochs contributing to analysis. Dotted orange line indicates vehicle treatment epoch that acute and chronic administration epochs are compared.
3. Comparison between effects of THC or VEH on changes from vehicle injection in percent time spent in NREM sleep in male mice. No significant effects were detected.
4. Comparison between effects of THC or VEH on changes from vehicle injection in NREM bout duration in male mice. There is a significant treatment effect (F_treatment_(1,23) = 5.834; p = 0.0241), with posthoc tests finding differences between-groups during Last Injection-LON (p* = 0.0179).
5. Comparison between effects of THC or VEH on changes from vehicle injection in number of NREM bouts in male mice. There is a significant treatment effect (F_treatment_(1,23) = 7.398; p = 0.0122), with posthoc tests finding differences between-groups during Last Injection-LON (p* = 0.0428).
6. - (H) Same metrics as (C-D) but for REM sleep in male mice. There were no significant effects detected for percent time in REM (F) or REM bout duration (G). A significant effect of treatment was detected (F_treatment_(1,22) = 9.312; p = 0.0059), but posthoc tests did not detect between or within group effects.
7. - (N) Same metrics as (C)-(H) but in female mice. A significant interaction for REM bout duration was detected (F_photoperiod x treatment_(1,18) = 7.939; p = 0.0114), but posthoc tests did not detect between or within group effects.

## Supplemental Figure 5


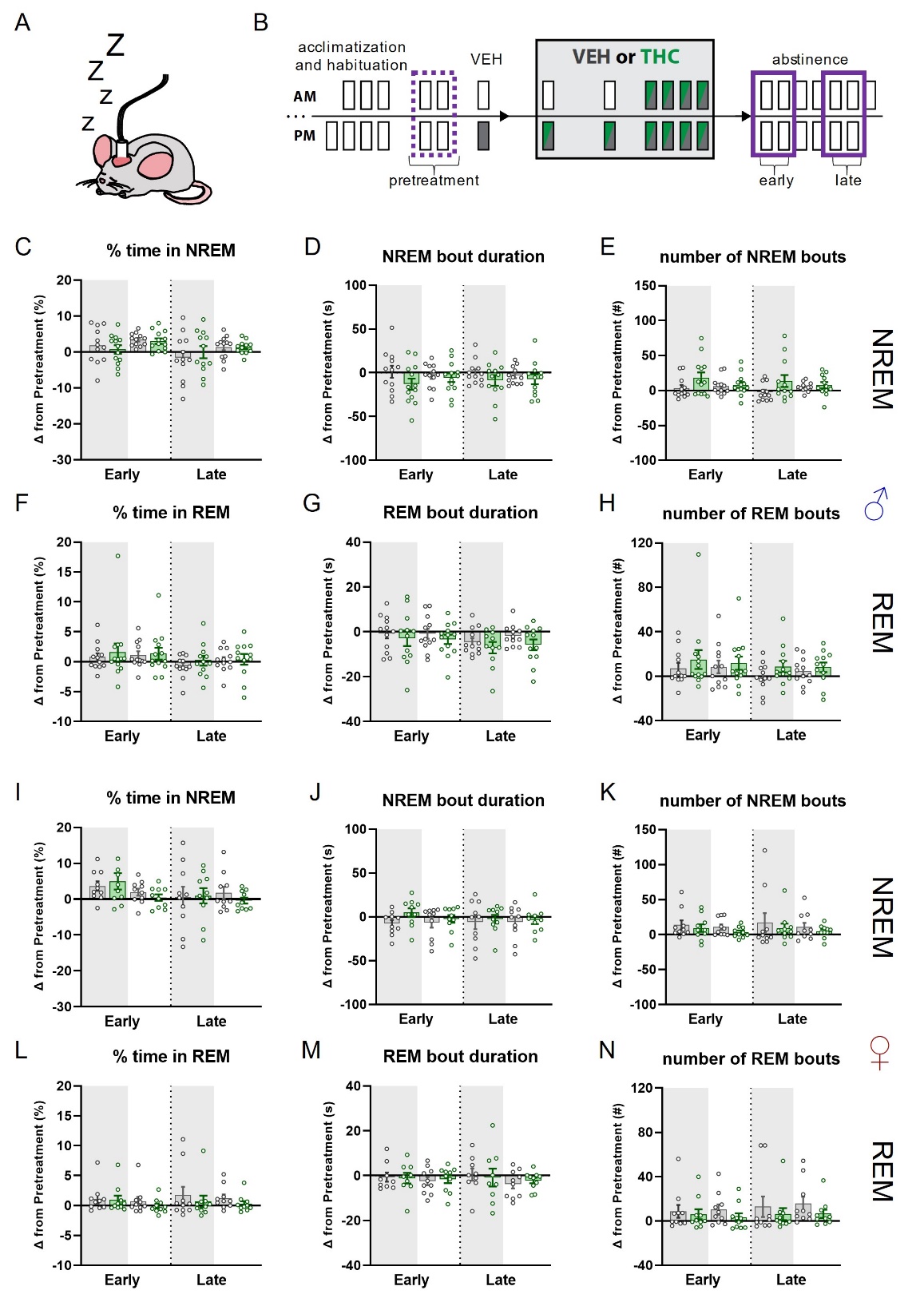


### Effects of chronic low dose (1.0 mg/kg) THC administration on sleep in male and female mice during early and late abstinence.

1. Cartoon illustrating chronically tethered mouse during epochs where no injection is given, i.e., pretreatment and abstinence.
2. Timeline illustrating treatment regimen and epochs contributing to analysis. Dotted purple line indicates average of two pretreatment epochs that average of days 1 and 2 of early and days 5 and 6 of late abstinence are compared.
3. - (N) No significant effects were detected in either.

## Supplemental Figure 6


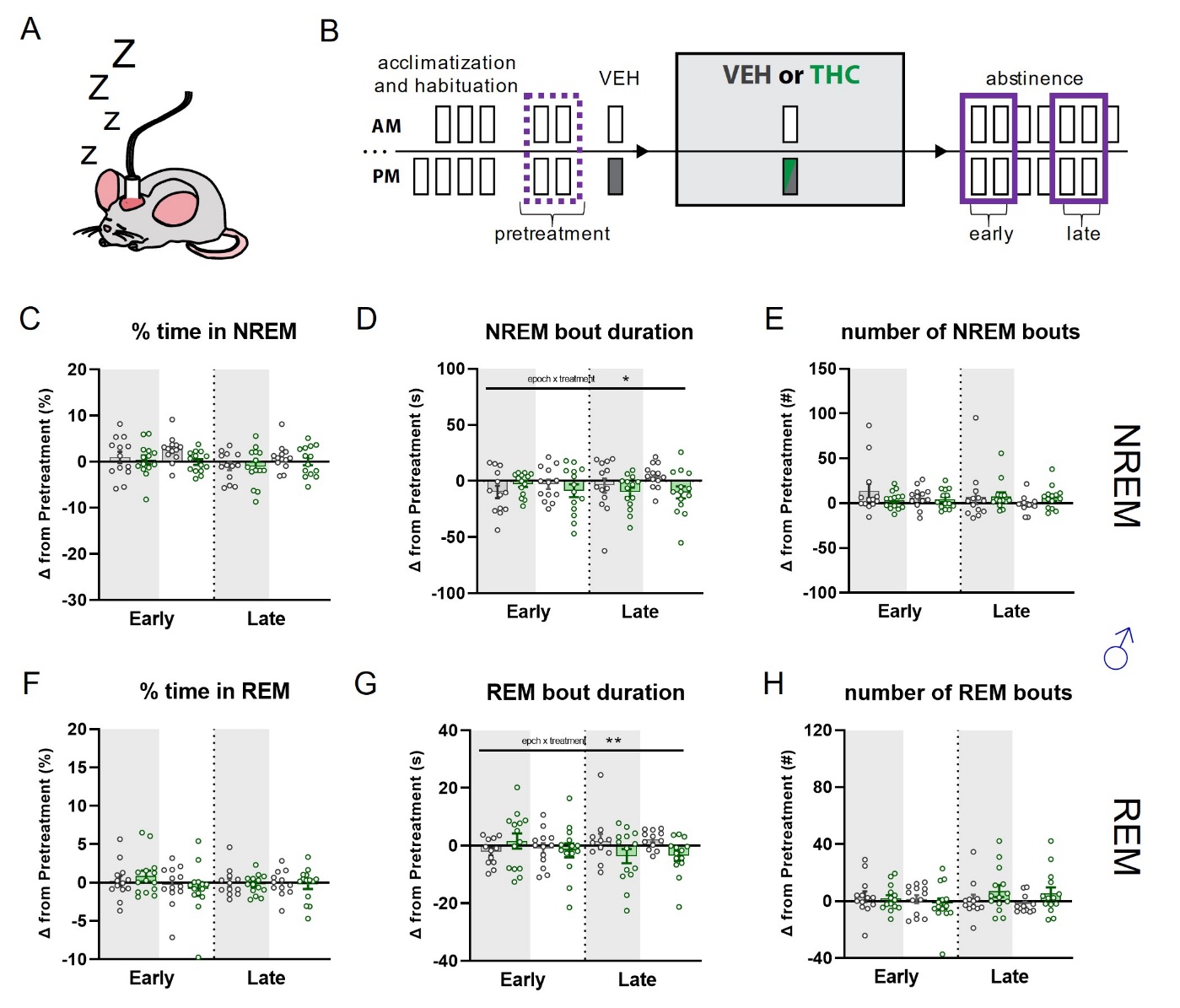


### Effects acute THC administration on sleep in male mice during early and late abstinence.

1. Cartoon illustrating chronically tethered mouse during epochs where no injection is given, i.e., pretreatment and abstinence.
2. Timeline illustrating treatment regimen and epochs contributing to analysis. Dotted purple line indicates average of two pretreatment epochs that average of days 1 and 2 of early and days 5 and 6 of late abstinence are compared.
3. - (H) NREM and REM metrics. Significant interaction effects were detected for bout duration in NREM (D; F_epoch x treatment_(1,25) = 5.388; p = 0.0287) and REM (G; F_epoch x treatment_(1,25) = 7.828; p = 0.0098). Posthoc tests for these metrics did not reveal any significant between or within group differences.

## Supplemental Figure 7


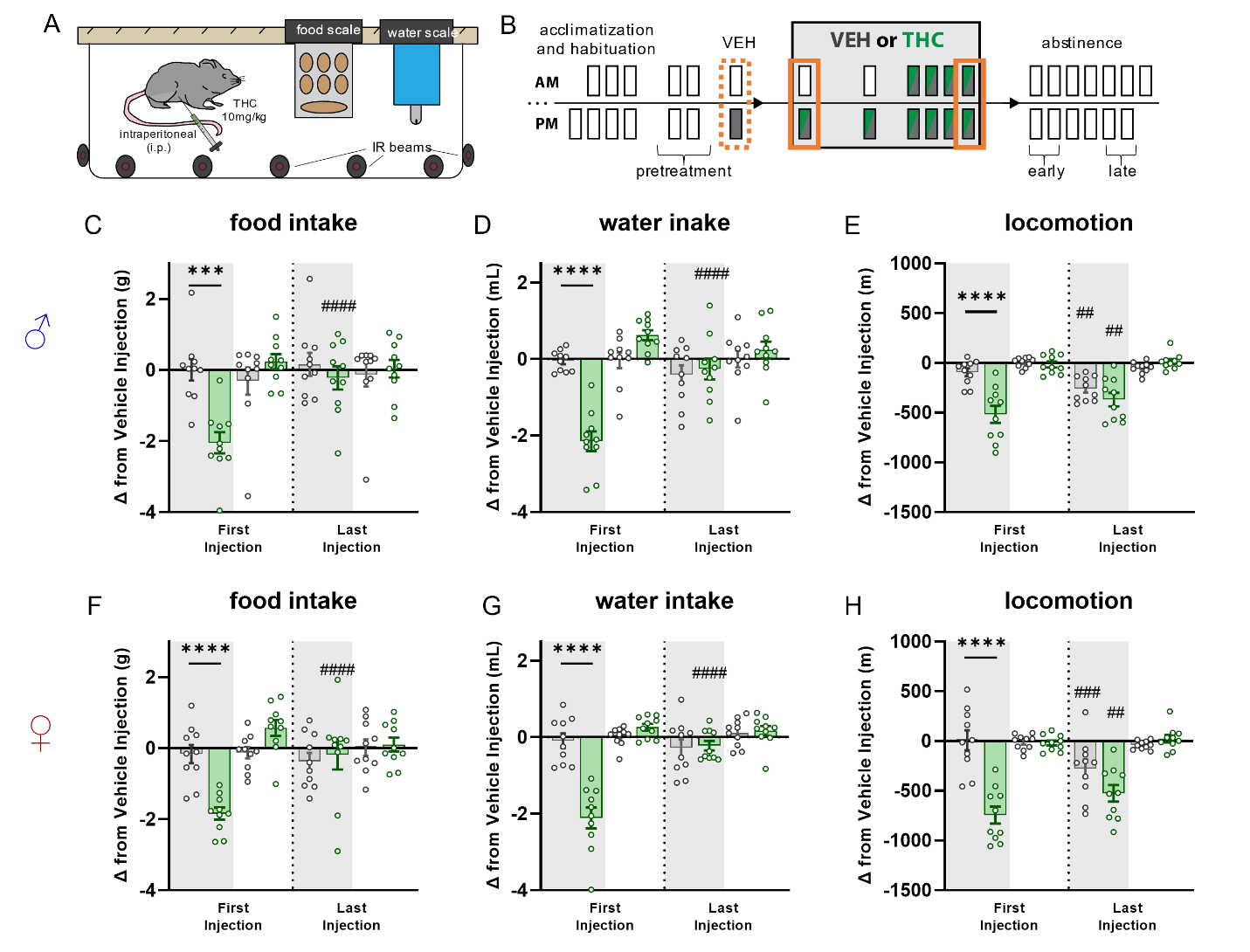


### Measurements of changes in food and water intake, and locomotion, during first and last THC injections.

1. Cartoon illustrating home-cage intake and locomotion monitoring platform.
2. Timeline illustrating treatment regimen and epochs contributing to analysis. Dotted orange line indicates vehicle treatment epoch that acute and chronic administration epochs are compared.
3. Comparison between effects of THC or VEH on changes from vehicle injection in food intake in male mice. There is a significant interaction effect (F_epoch x treatment_(1,18) = 11.37; p = 0.0034), with post-hoc tests finding differences between-groups during First Injection-LOFF (p*** = 0.0007) and within group differences for THC treated mice during Last Injection LOFF (p^####^ = <0.0001).
4. Comparison between effects of THC or VEH on changes from vehicle injection in water intake in male mice. There is a significant interaction effect (F_epoch x treatment_(1,18) = 14.43; p = 0.0013), with post-hoc tests finding differences between-groups during First Injection-LOFF (p**** = <0.0001) and within group differences for THC treated mice during Last Injection LOFF (p^####^ = <0.0001).
5. Comparison between effects of THC or VEH on changes from vehicle injection in locomotion in male mice. There is a significant treatment effect (F_treatment_(1,18) = 8.222; p = 0.0102), with post-hoc tests finding differences between-groups during First Injection-LOFF (p**** = <0.0001) and within group differences for THC treated (p^##^ = 0.0043) and VEH treated (p^##^ = 0.0015) mice during Last Injection LOFF.
6. Comparison between effects of THC or VEH on changes from vehicle injection in food intake in female mice. There is a significant interaction effect (F_epoch x treatment_(1,18) = 4.964; p = 0.0389), with post-hoc tests finding differences between-groups during First Injection-LOFF (p**** = <0.0001) and within group differences for THC treated mice during Last Injection LOFF (p^####^ = <0.0001).
7. Comparison between effects of THC or VEH on changes from vehicle injection in water intake in female mice. There is a significant treatment effect (F_treatment_(1,18) = 18.517; p = 0.0092), with post-hoc tests finding differences between-groups during First Injection-LOFF (p**** = <0.0001) and within group differences for THC treated mice during Last Injection LOFF (p^####^ = <0.0001).
8. Comparison between effects of THC or VEH on changes from vehicle injection in locomotion in female mice. There is a significant treatment effect (F_treatment_(1,18) = 14.03; p = 0.0014), with post-hoc tests finding differences between-groups during First Injection-LOFF (p**** = <0.0001) and within group differences for THC treated (p^##^ = 0.0026) and VEH treated (p^###^ = 0.0002) mice during Last Injection LOFF.

## Supplemental Figure 8


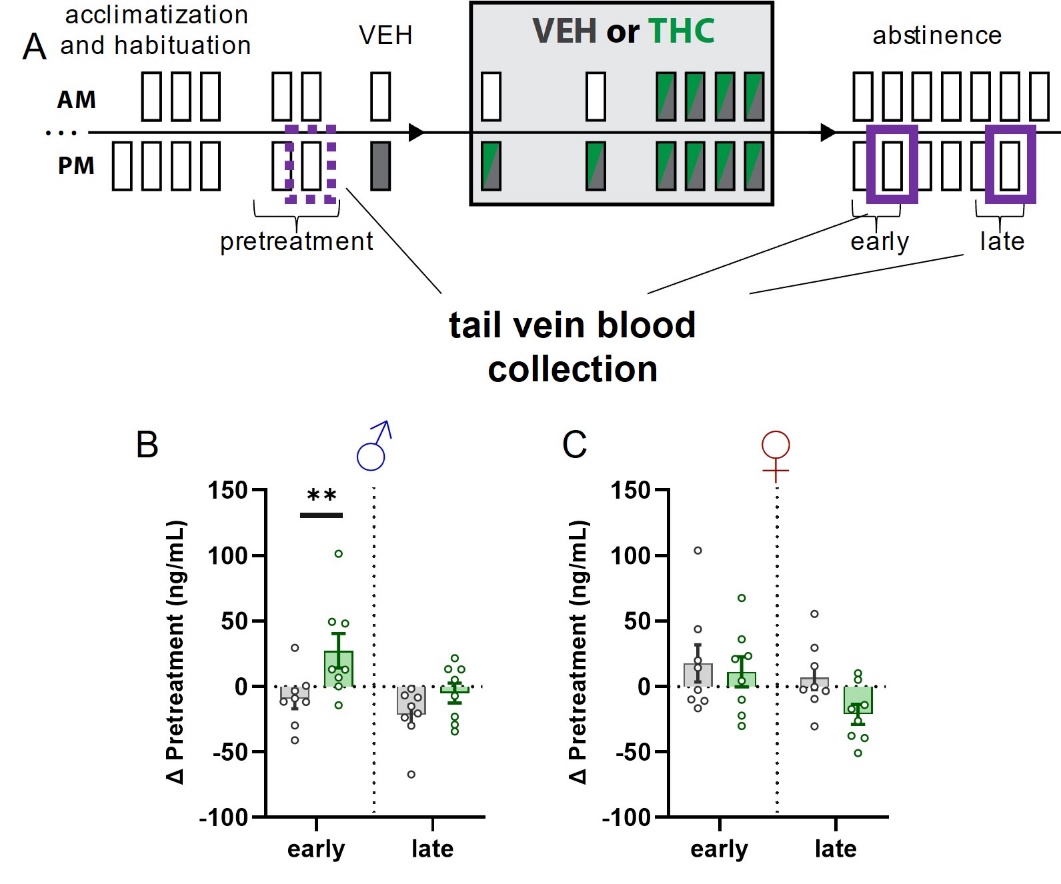


### Effect of chronic THC treatment on circulating plasma corticosterone levels in male in female mice during Early and Late abstinence.

1. Timeline illustrating treatment regimen and epochs contributing to analysis. Dotted purple line indicates pretreatment sample that Early and Late abstinence samples are compared.
2. Change in plasma CORT from pretreatment levels in Early and Late abstinence in male mice. A significant effect of treatment was detected F_treatment_(1,14) = 6.115; p = 0.0332) with posthoc tests finding between-group differences in Early abstinence (p** = 0.0168).
3. Same as (B) but for female mice. No significant effects were detected.

## Supplemental Table 1


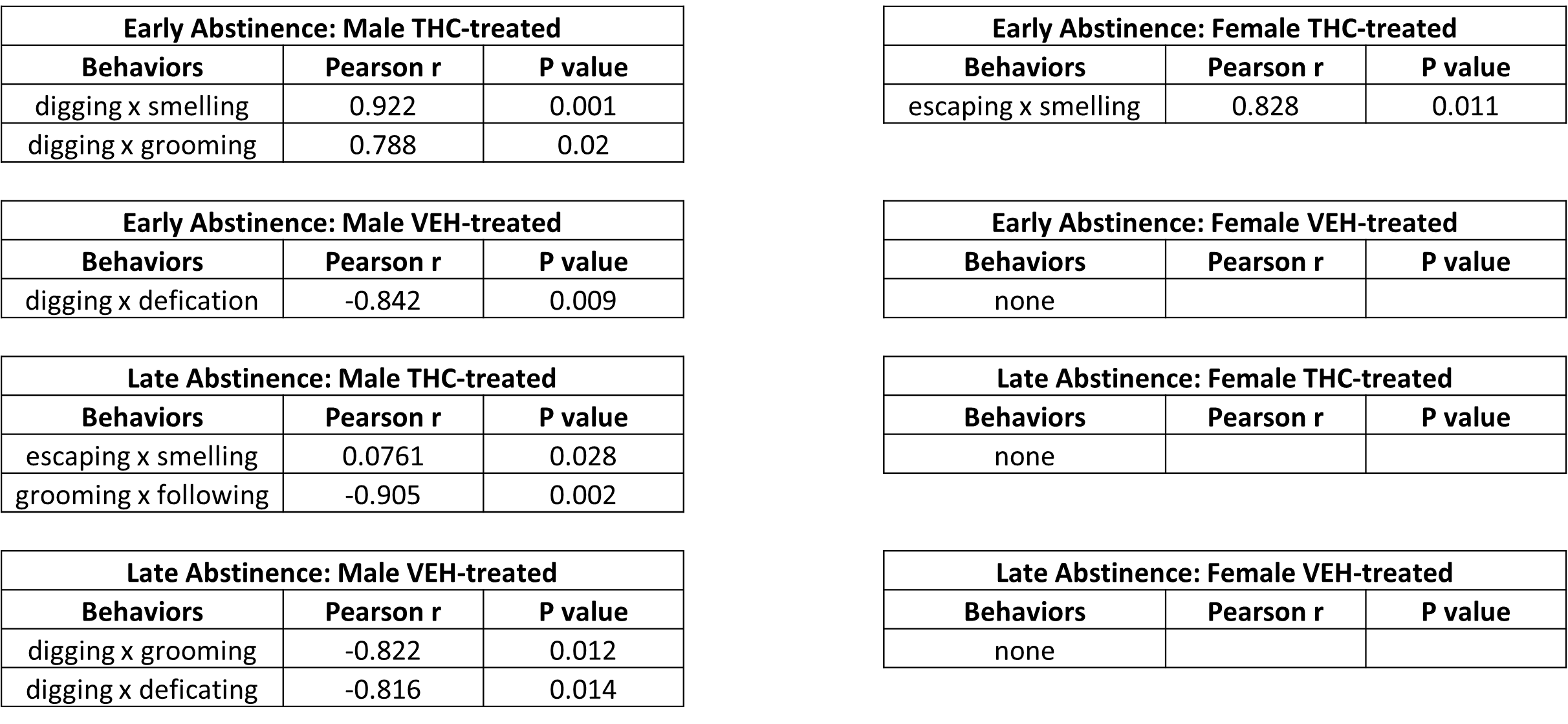


### Correlated behaviors during the bottle brush test

A correlation matrix was calculated between each pair of behaviors examined (see text for full list of behaviors). Only the behaviors indicated in the tables above had significant (p >0.05) correlations (Pearson r value).

6. REFERENCES

1. Pava, M.J., A. Makriyannis, and D.M. Lovinger, *Endocannabinoid Signaling Regulates Sleep Stability.* PLoS One, 2016. **11**(3): p. e0152473.

2. Abrahao, K.P., M.J. Pava, and D.M. Lovinger, *Dose-dependent alcohol effects on electroencephalogram: Sedation/anesthesia is qualitatively distinct from sleep.* Neuropharmacology, 2020. **164**: p. 107913.

3. Gervasoni, D., et al., *Global forebrain dynamics predict rat behavioral states and their transitions.* J Neurosci, 2004. **24**(49): p. 11137-47.

4. Bastianini, S., et al., *SCOPRISM: a new algorithm for automatic sleep scoring in mice.* J Neurosci Methods, 2014. **235**: p. 277-84.

5. Diniz Behn, C.G., et al., *Abnormal sleep/wake dynamics in orexin knockout mice.* Sleep, 2010. **33**(3): p. 297-306.

6. Weber, F., et al., *Control of REM sleep by ventral medulla GABAergic neurons.* Nature, 2015. **526**(7573): p. 435-8.

7. Riittinen, M.L., et al., *Impoverished rearing conditions increase stress-induced irritability in mice.* Dev Psychobiol, 1986. **19**(2): p. 105-11.

8. Lagerspetz, K. and R. Portin, *Simulation of cues eliciting aggressive responses in mice at two age levels.* J Genet Psychol, 1968. **113**(1st Half): p. 53-63.

9. Kimbrough, A., et al., *CRF1 Receptor-Dependent Increases in Irritability-Like Behavior During Abstinence from Chronic Intermittent Ethanol Vapor Exposure.* Alcohol Clin Exp Res, 2017. **41**(11): p. 1886-1895.
